# A digital twin framework for predicting and simulating type 2 diabetes onset using retrospective lifestyle data

**DOI:** 10.3389/fdgth.2026.1710829

**Published:** 2026-03-05

**Authors:** Mahreen Kiran, Ying Xie, Graham Ball, Rudolph Schutte, Nasreen Anjum, Barbara Pierscionek

**Affiliations:** 1 Faculty of Health, Medicine and Social Care, Anglia Ruskin University, Chelmsford, United Kingdom; 2 Faculty of Business and Management, Cranfield University, Cranfield, United Kingdom; 3 Intelligent Omics Ltd., Nottingham, United Kingdom; 4 School of Computing, University of Portsmouth, Portsmouth, United Kingdom

**Keywords:** artificial intelligence (AI), casual interference, Cox regression, diabetes prediction, digital twin, machine learning, survival analysis, type 2 diabetes mellitus (T2DM)

## Abstract

**Introduction:**

Type 2 Diabetes Mellitus (T2DM) is a rising global health concern, heavily influenced by modifiable lifestyle and psychosocial factors. However, most predictive tools focus on biomedical markers and rely on real-time data from wearables or electronic health records, limiting their scalability in resource-constrained settings. This study presents a novel digital twin (DT) framework that uses retrospective lifestyle, behavioral, and psychosocial data to forecast T2DM onset and simulate the estimated effects of preventive interventions.

**Methods:**

Data were drawn from 19,774 participants in the UK Biobank cohort, followed for up to 17 years. A penalized Cox proportional hazards model was employed to estimate individual time-to-event risk trajectories based on 90 candidate predictors. Predictors were selected through univariate screening, multicollinearity assessment, and variance filtering, yielding a final model with 14 significant variables. Causal inference techniques, including directed acyclic graphs (DAGs) and counterfactual simulations, were used to explore intervention effects on disease progression.

**Results:**

The model demonstrated strong predictive performance (C-index = 0.90, SD = 0.004). Psychosocial stressors such as loneliness, insomnia, and poor mental health emerged as strong independent predictors and were associated with estimated increases in absolute T2DM risk of approximately 35 percentage points individually and nearly 78 percentage points when combined, under the modeled assumptions. These effects were partly reinforced through diet, with high intake of processed meat, salt, and sugary cereals acting as risk amplifiers within the modeled causal pathways. Cheese intake was protective overall, but its estimated benefit was attenuated under psychosocial stress, where reduced consumption produced a small, directionally harmful mediation effect. Counterfactual simulations suggested that improvements in psychosocial conditions could reduce estimated T2DM risk by approximately 11.6 percentage points within the modeled cohort, with protective dietary patterns such as cheese consumption re-emerging as psychosocial stress was alleviated. The model also revealed pronounced ethnic disparities, with South Asian, African, and Caribbean participants exhibiting significantly higher estimated risk than White counterparts within this cohort. These findings highlight the potential of integrated, stress-informed prevention strategies that address both psychosocial and dietary pathways.

**Conclusion:**

This study introduces a transparent, simulation-enabled DT framework for estimating T2DM risk and exploring behavioral intervention scenarios without reliance on real-time data streams. It enables interpretable, personalized prevention planning and supports exploration of scalable deployment in public health, particularly in underserved or low-infrastructure environments. The integration of psychosocial and lifestyle data represents an important step toward more equitable and behaviorally informed digital health solutions.

## 1 Introduction

Type 2 Diabetes Mellitus (T2DM) is a chronic and progressive metabolic disorder marked by persistently elevated blood glucose levels, or hyperglycemia. According to the International Diabetes Federation (IDF), T2DM remains one of the most pressing global public health concerns, currently affecting over 537 million people worldwide. The IDF projects that this number will rise sharply to 643 million by 2030 and further to 783 million by 2045 [1]. If undiagnosed or poorly managed, T2DM can lead to a range of severe health outcomes, including kidney failure, vision loss, limb amputations, and increased mortality risk [2].

While genetic factors such as variants in the TCF7L2, FTO, and PPARG genes contribute to the development of T2DM [3, 4], there is strong evidence that modifiable lifestyle factors play a more dominant role in its onset and progression [5, 6]. These are behaviors or conditions that individuals can change through personal action or public health interventions, including physical inactivity, unhealthy dietary habits, excessive body weight (obesity), and chronic psychosocial stress. These factors contribute to metabolic dysfunction and are strongly associated with long-term complications such as cardiovascular disease, kidney damage, and stroke [7, 8].

Emerging evidence also underscores alcohol consumption as a vascular risk factor, with recent UK Biobank analyses showing positive linear associations between alcohol intake and arterial stiffness, challenging earlier assumptions of protective effects at moderate levels [9]. Additionally, chronic psychosocial stress, which may arise from financial hardship, social isolation, job related pressure, or caregiving responsibilities, is increasingly linked to elevated T2DM risk. Such stressors, together with mental health issues such as depression, anxiety, and sleep disturbances, can negatively affect daily routines, impair self regulation, and disrupt biological processes. These disruptions contribute to insulin resistance and poor glycemic control through both behavioral and physiological pathways [10–13].

### 1.1 Problem statement

Despite this growing understanding, current risk prediction models and clinical decision tools for T2DM remain predominantly centered on conventional biomedical indicators such as body mass index (BMI), age, fasting glucose, and blood pressure [14, 15]. This narrow clinical focus often overlooks the interconnected behavioral and emotional factors that precede and shape disease onset, creating a significant gap in the design of prevention strategies [16].

To improve prediction and personalization in chronic disease management, technologies such as artificial intelligence (AI), machine learning (ML), and digital twins (DTs) have gained significant attention in recent years. Research is currently being conducted on DTs to explore the possibility of generating dynamic models and simulations of human physiology, with the goal of enhancing patient care and treatment options [17–19]. These approaches show promise, but they also face important limitations [20–23]. Many AI and ML models operate as “black boxes.” They can make accurate predictions but often do not explain how or why certain outcomes occur, which limits their interpretability in clinical settings [24]. While some studies [10–13] have begun to incorporate psychosocial factors such as stress, loneliness, and mental health, these variables remain underrepresented in many mainstream models. As a result, the broader influence of behavioral and emotional factors on outcomes like T2DM is often overlooked or insufficiently modeled, despite growing evidence of their clinical relevance.

DT systems, which are virtual models that replicate individual health profiles to simulate disease progression and intervention effects, offer a promising approach for advancing personalized and preventive medicine. By integrating diverse health data, DTs enable clinicians to test ”what-if” scenarios and tailor care to individual needs [25, 26]. However, most existing DT implementations rely heavily on real-time data streams from wearables, biosensors, or electronic health records (EHRs), which presents significant limitations. These include the need for continuous data acquisition, costly technological infrastructure, and substantial concerns around data privacy and interoperability [27]. Such constraints restrict the scalability and adoption of DT systems, especially in low-resource or decentralized healthcare settings.

### 1.2 Proposed solution

To address the limitations of existing prediction models and DT systems for T2DM, this study introduces a novel DT prototype that operates entirely on historical behavioral and psychosocial data and does not require real-time data streams or specialized sensors, making it suitable for deployment in settings with limited technical infrastructure. By utilizing existing datasets, such as the UK Biobank [28], the prototype supports population-level scalability while retaining the capacity for individualized simulation. This positions the framework as a cost-effective and widely accessible solution for early-stage prevention, particularly in public health settings and underserved communities.

Unlike traditional models that focus on physiological markers (BMI, blood glucose, and age etc.,) the framework presented in this study includes psychosocial risk factors such as loneliness, sleep disturbances (e.g., insomnia), and depressive symptoms. These variables are not only included in survival analysis for outcome prediction but are also positioned as causal mediators and moderators within the disease pathway, offering a richer and more behaviorally contextualized understanding of T2DM risk.

To maintain transparency and clinical relevance, the system uses penalized Cox regression [29] for survival modeling and incorporates causal inference methods such as backdoor adjustment [30] and mediation analysis. This allows clinicians and researchers to trace how modifiable risk factors influence outcomes through biologically and behaviorally plausible pathways, enhancing model credibility, clinical trust, and interpretability.

Beyond predictive accuracy, this study lays the groundwork for a new class of simulation-enabled, behavior-aware DT systems for chronic disease prevention. It offers actionable insights for a broad spectrum of stakeholders: clinicians gain tools for tailored risk management, researchers benefit from a validated framework for behavioral modeling, and policymakers are empowered with scalable strategies for public health intervention, particularly in settings where real-time data infrastructure is limited. Finally, the proposed DT system was validated using a series of techniques recommended by Sharma et al. [31]. These included placebo tests, subset sampling, bootstrap refutation tests, and sensitivity analysis to detect hidden confounding, thereby ensuring the robustness of the findings.

Together, these elements contribute to a novel, simulation enabled DT prototype capable of generating personalized, interpretable, and clinically actionable prevention strategies for T2DM, particularly in environments where real-time data capture is not feasible.

## 2 Related work, research gap and key contributions

DTs have demonstrated considerable potential for enhancing patient monitoring, risk stratification, and individualized treatment planning [32, 33]. While effective in high resource or clinical settings, these implementations face significant challenges related to cost, scalability, and privacy, which hinder their broader deployment, particularly in community level or resource limited environments.

### 2.1 DT-driven precision nutrition and remission strategies

One of the most advanced applications of DTs in T2DM lies in precision nutrition. Shamanna et al. [34, 35] demonstrated that integrating continuous glucose monitoring data with dietary inputs improved glycemic outcomes, reduced BMI, and decreased reliance on diabetes medication. Their Twin Precision Nutrition (TPN) and Twin Precision Treatment (TPT) platforms supported rapid and sustained metabolic benefits [36], and introduced a structured, DT-guided seven-stage remission model [37].

However, these systems rely on continuous biosignal input and clinical oversight, which limits their use in preventive or low infrastructure contexts. In contrast, our study develops a retrospective, data driven DT prototype that simulates disease risk and intervention outcomes using behavioral, dietary, and psychosocial variables, without requiring real-time inputs. This enhances scalability and enables deployment in community level prevention programs.

### 2.2 Digital twins for comorbidity management

DT applications are also expanding to address multimorbidity, particularly in patients with T2DM and related conditions like hypertension and cardiovascular disease. Shamanna et al. [38] showed that DT-enabled systems reduced antihypertensive medication use while improving metabolic and cardiovascular markers. However, like their earlier platforms, these systems remain tied to real-time biosensor data and frequent clinical engagement, limiting broader applicability.

### 2.3 Offline and simulation-driven DT models

Recent work has explored DT frameworks that do not require real-time monitoring. For example, Silfvergren et al. [39] modeled glycemic responses to macronutrient intake using trial data, and Vaskovsky et al. [40] developed an adaptive food recommendation system based on genetic predisposition. While promising, these studies focus primarily on nutrition and do not fully incorporate behavioral simulation, causal reasoning, or long-term disease forecasting.

### 2.4 Ethical, regulatory, and interpretability considerations

As DTs become more prevalent in health systems, concerns around transparency, explainability, and data governance grow. Prior research [41] stresses the importance of interpretability in digital health. Our study addresses this by using transparent statistical and causal inference methods that yield clinically meaningful, traceable outputs. This supports ethical alignment and enhances trust in clinical and policy decision-making.

#### 2.4.1 Research gap

Despite substantial progress in the application of digital twins to diabetes care, several key limitations remain under-explored:

- **Dependence on real-time data:** Most existing DT systems rely on continuous biosignal input from wearables or EHRs, making them impractical for low-resource or decentralized healthcare settings.
- **Underutilization of psychosocial variables:** Critical behavioral and psychosocial factors, such as loneliness, insomnia, and mental health history, are rarely integrated into DT frameworks despite their established role in influencing T2DM risk.
- **Limited use of causal modeling:** Many current DT approaches rely on associative ML models, which limits their capacity to simulate personalized interventions or model causal pathways.
- **Focus on disease management over prevention:** The majority of DT systems are reactive, geared toward disease monitoring or treatment optimization rather than early risk prediction and preventive intervention.

#### 2.4.2 Key contributions

To address the identified gaps, this study makes the following contributions:

- **Retrospective, scalable DT prototype:** A framework is introduced that operates entirely on historical behavioral and psychosocial data, eliminating the need for real-time sensors or clinical infrastructure.
- **Behaviorally and psychosocially enriched modeling:** The DT integrates underused psychosocial variables, such as loneliness, insomnia, and depressive symptoms, as core predictors of T2DM risk.
- **Causal inference-driven simulation:** By applying methods such as backdoor adjustment and mediation analysis, the system supports counterfactual simulations to evaluate how modifiable behaviors influence disease onset.
- **Transparent, time-aware risk forecasting:** Using penalized Cox regression, the model delivers interpretable time-to-event predictions that enhance clinical trust and enable personalized prevention planning.
- **Validated, prevention-oriented design:** The framework is validated using cross validation, placebo testing, and sensitivity analysis, and is positioned for deployment in public health and low resource environments, thereby expanding the scope of DTs from clinical management to preventive care.

## 3 Proposed digital twin framework

**Figure 1:**
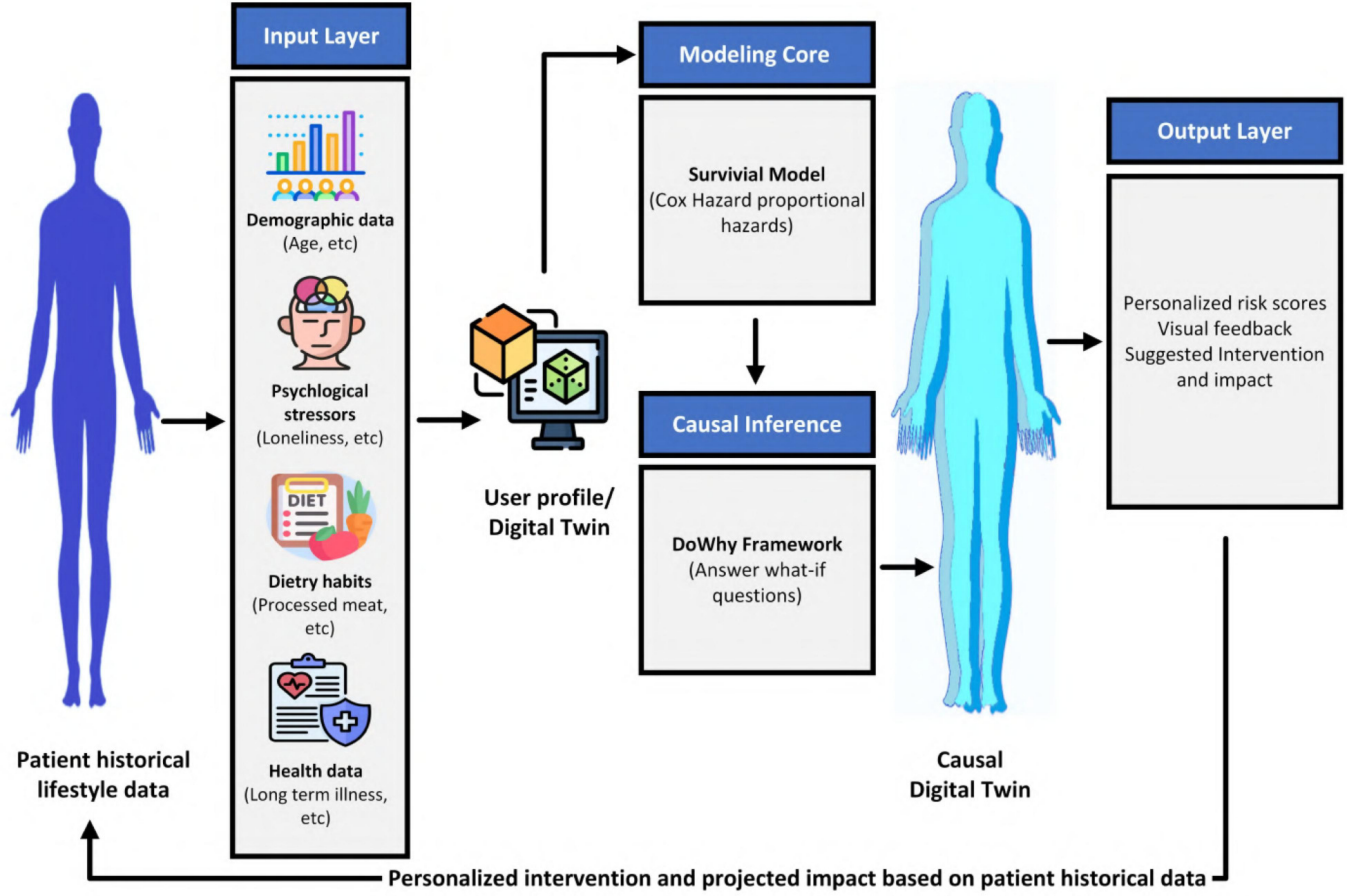
Digital Twin prototype framework for T2DM prediction and simulation: The architecture combines retrospective lifestyle data preprocessing, survival modeling using a penalized Cox proportional hazards model, and causal inference via the DoWhy framework. It enables both disease risk prediction and simulation of personalized interventions by estimating individual risk trajectories and recalculating risk scores under hypothetical “what-if” scenarios.

Figure 1 illustrate the system architecture and workflow of DT framework. The framework is organized into four interconnected layers: input, survival modeling, causal inference, and output. Together, these components enable both risk prediction and intervention simulation using retrospective lifestyle data.

1. **Input layer:** This layer sources and preprocesses retrospective lifestyle, dietary, demographic, and psychosocial data from the UK Biobank. Unlike conventional digital twins that depend on continuous monitoring devices or clinical biomarkers, this prototype deliberately excludes real-time data streams. The aim is to build a scalable and cost-effective twin that can function in community and low-resource settings (Sections 5, 6).
2. **Survival Modeling Core:** A penalized Cox proportional hazards model is employed to estimate individualized time-to-event trajectories for T2DM onset. This allows the twin to not only assign risk levels but also forecast when an individual is likely to develop the condition. The model integrates behavioral and psychosocial predictors alongside demographic variables, producing transparent and interpretable hazard ratios (Section 7).
3. **Causal inference and simulation:** To move beyond statistical association, the system incorporates causal reasoning through domain-informed directed acyclic graphs and counterfactual analysis. Using the DoWhy framework, the DT simulates ’what if’ scenarios. For example, it estimates how a reduction in loneliness, improved sleep, or decreased processed food intake would alter an individual’s diabetes risk. This transforms the DT into a dynamic simulation tool rather than a static predictor (Section 9).
4. **Output layer:** The final stage translates predictions and simulations into actionable results. It generates personalized risk scores, stratifies individuals into risk groups, and recalculates these outcomes under hypothetical interventions. In this way, the DT offers both risk forecasting and prevention-oriented recommendations tailored to individual and population profiles (Section 10).

In summary, the proposed DT framework integrates four sequential layers: input, survival modeling, causal inference, and output. Retrospective lifestyle and psychosocial data are first processed in the input layer, then modeled in the survival core to generate individualized risk trajectories. These outputs feed into the causal inference engine, which embeds predictors within a domain-informed causal graph to simulate counterfactual interventions. Finally, the output layer translates both predictive and simulated results into actionable insights, including risk scores, stratified risk groups, and prevention-oriented recommendations. This design ensures the DT operates not only as a predictive tool but also as a dynamic simulation system capable of supporting personalized and population-level diabetes prevention strategies.

## 4 Research methodology

**Figure 2:**
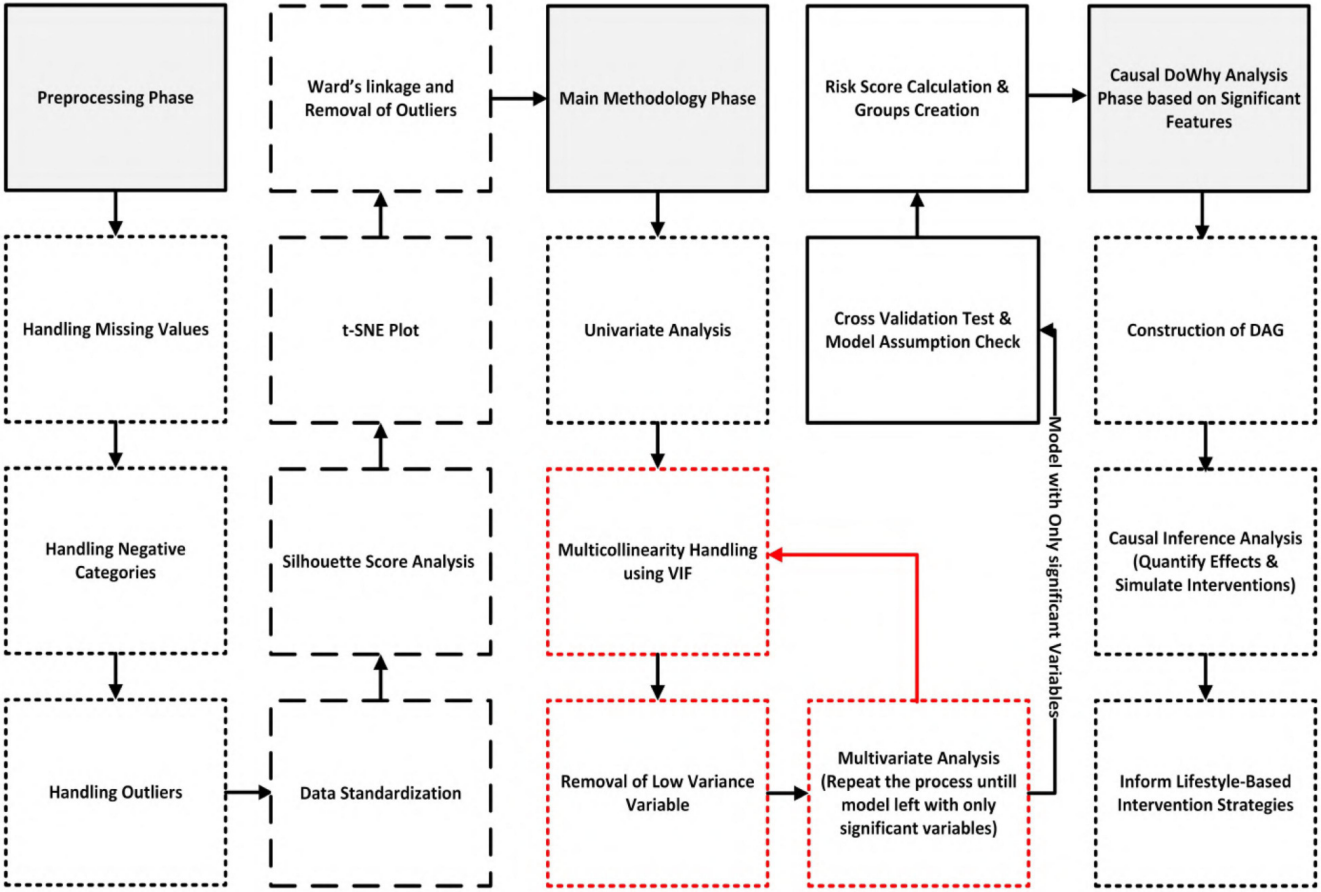
A schematic overview illustrating preprocessing, clustering, multivariate survival modeling, and causal inference steps leading to risk profiling and intervention simulation.

Figure 2 shows a step-by-step schematic of each analytical phase, spanning from data selection and preprocessing through to causal simulation and intervention planning. The methodology is organized into four main stages:

- **Dataset preparation:** UK Biobank data were filtered using strict inclusion criteria to support time-to-event analysis (Section 5).
- **Preprocessing and feature selection:** Standardization, imputation, and outlier exclusion were followed by univariate screening and multicollinearity checks (Section 6).
- **Survival modeling:** A multivariate Cox proportional hazards model was used to analyze time to T2DM diagnosis with right censoring and irregular follow-up; its semi-parametric form leaves the baseline hazard unspecified and yields efficiently estimated, clinically interpretable hazard ratios. To ensure robustness, proportional hazards diagnostics and C-index discrimination were assessed, and model stability was evaluated via cross-validation (Sections 7, 8).
- **Causal inference analysis:** Implementing domain-informed causal graphs and counterfactual analysis to estimate the effects of behavioral interventions (Section 9).

This approach enabled both individual risk prediction and detection of systemic behavioral disintegration. Cohesion shifts provided a key signal of emerging metabolic vulnerability, capturing how emotional, psychosocial, and demographic anchors in behavior erode prior to clinical diagnosis.

All model outputs, both predictive and causal, were rigorously validated to ensure credibility and generalizability, employing:

- **Cross-validation (C-index)** for predictive performance,
- **Placebo testing** to identify spurious associations,
- **Subset validation** and **bootstrap resampling** for sample robustness,
- **Sensitivity analysis** to evaluate the influence of unmeasured confounding.

These procedures confirm the model’s reliability, clinical utility, and ability to support personalized, evidence-based prevention strategies in both research and healthcare settings.

## 5 Dataset preparation

The foundational dataset was sourced from the UK Biobank [28], a large-scale, prospective cohort of over 500,000 participants aged 40–69 at baseline. It includes extensive behavioral, psychosocial, demographic, and clinical variables such as validated measures of depression, insomnia, loneliness, self-reported mental health, physical activity, sleep, and diet, critical for modeling behavioral pathways and simulating intervention outcomes.

**Figure 3:**
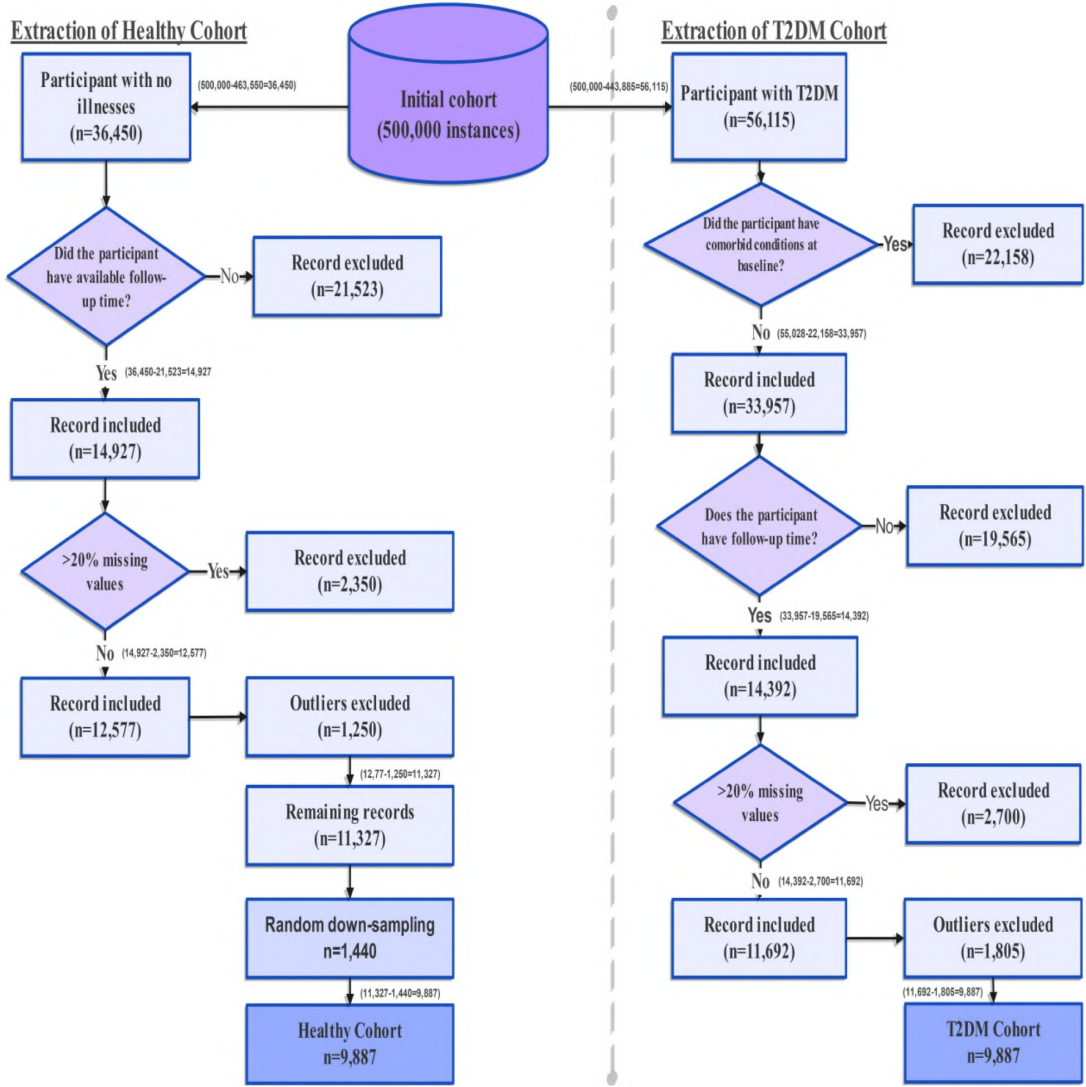
Flow diagram of participant selection from the UK Biobank cohort. Starting from approximately 500,000 records, inclusion and exclusion criteria were applied separately for T2DM and non-T2DM individuals. Records with comorbid illnesses, >20% missing data, and statistical outliers were excluded. Healthy individuals were randomly down-sampled to match the T2DM sample size. The final analytic dataset included 9,887 participants with T2DM and 9,887 healthy participants.

To construct a cohort suitable for time-to-event analysis, participants were conceptually separated into two groups based on disease status during follow-up: individuals who developed T2DM after baseline assessment (T2DM cohort) and individuals who remained non-diabetic throughout the observation period (Healthy cohort). The full workflow is illustrated in Figure 3.

### 5.1 Healthy stream

For the healthy stream, individuals with any recorded chronic disease at baseline were excluded using linked clinical records and self reported diagnoses from the UK Biobank. Disease status was identified using field **41,270**, which captures ICD-10 coded diagnoses reported across hospital records and participant medical history. This process excluded all participants with T2DM and other major long term conditions. Applying this criterion removed 463,550 participants, leaving 36,450 individuals free of diabetes and other chronic diseases at baseline, as shown in the left-hand branch of Figure 3. These participants formed the healthy cohort prior to follow up verification and further data quality filtering.

This exclusion strategy was designed to ensure that observed behavioral, dietary, and psychosocial differences could be attributed specifically to diabetes rather than to the presence of multiple chronic conditions. Non-diabetic illnesses, including cancer, cardiovascular disease, kidney disease, and chronic respiratory disorders, are known to independently alter lifestyle behaviors and psychological health through disease related symptoms, functional limitations, and treatment burden [42–44]. Retaining participants with such conditions would therefore introduce confounding effects unrelated to diabetes itself, complicating the interpretation of diabetes specific behavioral patterns and reducing internal validity [45, 46]. Restricting the healthy cohort to participants without chronic disease thus provided a robust reference group for examining behavioral changes associated with the onset of T2DM.

### 5.2 T2DM stream

For the T2DM stream, participants were identified based on the presence of at least one ICD-10 diagnosis code E11.0 to E11.9 in linked hospital inpatient records. This yielded 56,115 individuals with a recorded diagnosis of T2DM. Participants without any T2DM diagnosis, approximately 443,885 individuals, were excluded from this stream. To reduce confounding from non diabetes related illnesses, individuals with major chronic comorbid conditions were subsequently excluded, resulting in a final T2DM cohort of 33,957 participants prior to missing data and outlier exclusion, as illustrated in the right-hand branch of Figure 3.

Eligibility further required the availability of sufficient follow-up information to define either an event time or a censoring time. Participants who remained free of T2DM throughout follow-up were treated as censored observations. For these individuals, follow-up time was defined from the baseline assessment to the most recent subsequent assessment or linked record confirming continued non-diabetic status, as indicated by UK Biobank field **53**. Participants without any follow-up assessment or linked hospital record confirming non-diabetic status were excluded due to the inability to define a valid censoring time. After applying this criterion, 14,927 healthy participants had valid follow-up time.

For participants who developed T2DM during follow-up, incident disease was identified using ICD-10 codes E11.0–E11.9 recorded in UK Biobank linked hospital inpatient data [47]. The date of first recorded diagnosis, captured in UK Biobank field **130,708**, was used to define the event time. Follow-up time for these individuals was calculated as the interval between baseline assessment and this first diagnosis date, provided the diagnosis occurred after baseline. This approach yields valid survival times even for individuals without repeat assessment visits, as diagnosis dates are obtained through hospital linkage. After applying this rule, 14,392 T2DM participants had a valid event time. Together, these definitions ensured consistent and unbiased estimation of time-to-event outcomes across the cohort. Related methodological principles for cohort construction and survival eligibility using UK Biobank data have been described in our prior work [48] and are referenced here for methodological context, while all dataset-specific decisions are fully documented in the present study. The resulting sample size and number of observed events were sufficient to support stable time-to-event modeling relative to the final number of predictors included.

## 6 Data preprocessing and feature selection

To support the objectives of the Digital Twin framework, clinical biomarker variables such as blood glucose, glycated hemoglobin, and cholesterol were excluded from the predictor set. While these measures are clinically informative and commonly used in diabetes risk assessment [49], their inclusion would anchor the model to metabolic abnormalities that typically emerge later in the disease process. In contrast, the present study focuses on early risk forecasting and the simulation of preventive interventions using modifiable lifestyle and psychosocial factors that are observable prior to routine laboratory abnormalities.

After defining the predictor scope, data preprocessing steps were applied to the 14,927 healthy records and 14,392 T2DM records to ensure analytical stability and reproducibility within the Digital Twin framework. Categorical responses such as “Don’t know” and “Prefer not to answer” were recoded as missing values (NaN). Records with more than 20% missing data were excluded to balance data completeness with sample retention and to limit instability arising from extensive imputation, consistent with common practice in epidemiological and machine-learning analyses [50, 51]. Applying this threshold removed 2,350 records from the healthy group, leaving 12,557 individuals, and excluded 2,700 records from the T2DM group, resulting in 11,692 participants.

For the remaining observations, missing values were imputed using mode imputation for categorical variables, mean imputation for continuous variables, and the least frequent category for binary fields. Negatively coded responses (e.g., “−7” indicating “None of the above”) were recoded to preserve logical and ordinal structure across variables. Following missing data handling and imputation, additional preprocessing steps were undertaken to address structurally inconsistent records.

### 6.1 Outlier handling and detection

Outliers can distort multivariate relationships in behavioral health data and bias survival estimates. In this study, rather than adjusting extreme values, which can alter inter-variable dependencies and obscure higher-order behavioral structure [52–54], entire records flagged as structurally inconsistent were excluded. This full-case removal strategy preserves coherence across behavioral features and supports stable estimation in Cox proportional hazards models. Outlier detection was performed after missing-data filtering and prior to any supervised modeling to prevent data leakage and ensure valid survival inference.

#### 6.1.1 Outlier detection pipeline

A multi-step unsupervised approach was used to detect and remove structurally inconsistent data points, ensuring a robust dataset for analysis while balancing generalizability and internal validity.

**Figure 4:**
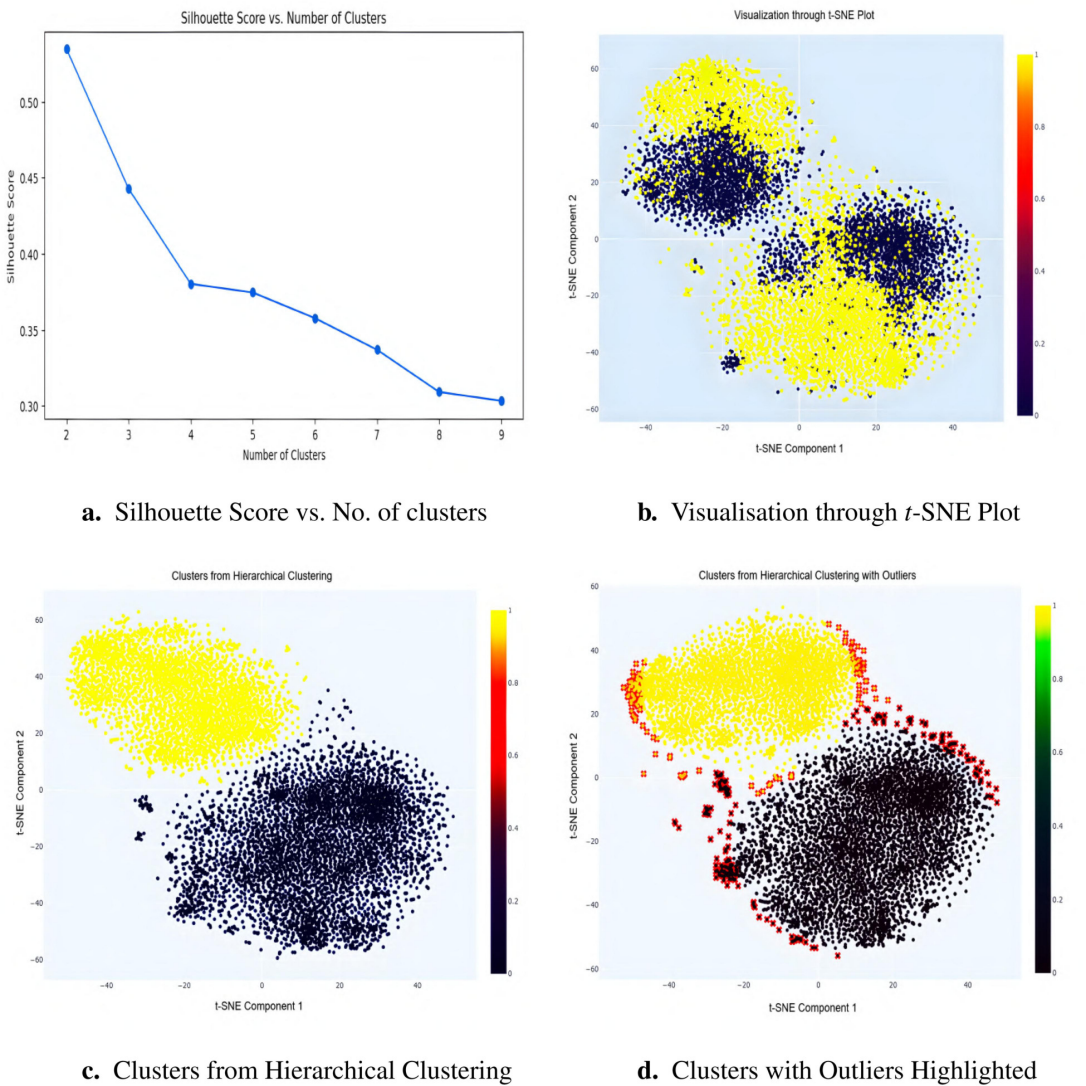
Silhouette score analysis **(a)** indicates optimal clustering at k=2. The *t*-SNE projection **(b)** visually separates these clusters, highlighting outliers at the data margins. Hierarchical clustering using Ward’s linkage **(c)** refines cluster integrity, while final visualization **(d)** reveals peripheral outliers (red) subsequently removed to enhance data quality and improve model robustness.

- **Data standardization:** All features were standardized to a mean of zero and standard deviation of one to ensure equal contribution, especially for algorithms like t-distributed Stochastic Neighbor Embedding (*t*-SNE) [55], which are sensitive to feature scale.
- **Optimal cluster selection via Silhouette score:** Silhouette analysis [56] was used to evaluate clustering quality, with the highest score at k=2, indicating optimal cluster separation (see Figure 4a).
- ***t*-SNE visualization:** *t*-SNE was applied to project the dataset into two dimensions using a perplexity of 40 and 5,000 iterations. It was chosen over principal component analysis (PCA) [57] because it more effectively preserved local neighborhood structures in the data used in this study, which facilitated clearer cluster separation and outlier detection. The resulting two-dimensional projection revealed two distinct clusters corresponding to the binary target variable, along with peripheral points that may represent outliers (see Figure 4b).
- **Hierarchical Clustering:** Ward’s linkage method [58] was used on the *t*-SNE components, producing well-separated clusters with minimized intra-cluster variance (see Figure 4c).
- **Outlier identification and removal:** Outliers were identified based on their Euclidean distance from the centroid of their respective clusters:
  - ***Centroid calculation:*** Centroids of each cluster in *t*-SNE space were computed.
  - ***Distance measurement:*** Euclidean distances between each data point and its cluster centroid were calculated.
  - ***Thresholding:*** Points beyond the 95th percentile of these distances were flagged as outliers.
  - ***Visualization and removal:*** Outliers were marked in red in Figure 4d and subsequently removed to avoid skewing the analysis.

This systematic pipeline ensured the removal of structurally inconsistent data points, resulting in improved cluster cohesion and enhanced analytical integrity. While this approach enhances internal validity and model fit, it does come with a trade-off in terms of external generalizability. Removing extreme data points may limit the representativeness of certain subpopulations. However, for time-to-event modeling and simulation purposes, this trade-off was considered acceptable to improve robustness and reduce model bias. Applying the pipeline removed an additional 11,327 outliers from the healthy cohort and 1,805 from the T2DM cohort, reducing the samples from 12,557 to 1,230 healthy participants and from 11,692 to 9,887 T2DM participants. These constituted the pre balanced cohorts used for subsequent survival analysis.

#### 6.1.2 Final study cohort

After validating follow-up times and completing missing data and outlier filtering, the Healthy and T2DM cohorts were advanced to the final stage of cohort construction. The healthy group remained moderately larger than the T2DM group. Although Cox proportional hazards models do not require balanced outcome groups, random down-sampling was applied to achieve matched sample sizes for computational balance and interpretability, without conditioning on predictor variables. This procedure altered the observed prevalence of T2DM but does not bias Cox model estimation, as inference is driven by time-to-event ordering and risk sets rather than marginal outcome proportions [59–61]. Key model estimates and performance metrics were consistent with analyses conducted on the full cohort, indicating robustness to this design choice. As shown in Figure 3, the final analytic dataset included 19,774 individuals, with 9,887 participants who were non-diabetic at baseline and subsequently developed T2DM during follow up (T2DM cohort), and 9,887 participants who remained non-diabetic across all available assessments (Healthy cohort). Time-to-event durations ranged from 1 to 17 years, providing sufficient longitudinal variability for robust and unbiased survival analysis. All preprocessing thresholds and parameter settings were specified a priori and applied uniformly, with no tuning based on outcome information.

## 7 Survival modeling and risk identification

This section outlines the Cox modeling pipeline, covering variable screening, multicollinearity control, proportional hazards (PH) testing, and model validation, followed by interpretation of significant predictors forming the basis for simulation and intervention in the DT system.

### 7.1 Cox model development and validation

A penalized Cox proportional hazards model [29] was employed to estimate T2DM onset risk. The model simultaneously identified significant predictors and generated time-sensitive risk estimates, enabling personalized simulation. The hazard function is given in Equation 1:

$$h(t|X)=h_{0}(t)\cdot \exp(\beta_{1}X_{1}+\beta_{2}X_{2}+\cdots +\beta_{p}X_{p})$$ (1)

where h(t∣X) is the hazard at time t, $h_{0}(t)$ is the baseline hazard, and $\exp(\beta_{i})$ represents the hazard ratio (HR) for predictor $X_{i}$.

#### 7.1.1 Univariate analysis

Before building the multivariate survival model, each predictor was first evaluated individually to screen for potential associations with T2DM onset. To do this, a univariate Cox proportional hazards regression was conducted for each variable in the dataset. This process involves modeling the time to T2DM onset as a function of a single predictor at a time, allowing for an independent assessment of each variable’s association with the outcome. It should be noted that univariate screening was used solely as an initial dimensionality reduction step and not as a criterion for final variable selection, which will be determined in the multivariate modeling stage.

##### 7.1.1.1 Variable encoding and significance testing

Variable types were handled according to their characteristics to ensure proper integration into the Cox regression model. Categorical predictors were encoded using dummy variables, with one category designated as the reference group and the remaining categories represented by binary indicators. Effects were interpreted relative to the reference category, and categorical predictors were retained if at least one associated dummy variable demonstrated statistical significance (p<0.05). Continuous variables were entered in their original numerical form to capture incremental effects on risk over time, while binary variables were included directly without further transformation.

##### 7.1.1.2 Variance filtering and multicollinearity control

To enhance the stability, interpretability, and overall quality of the final model, two critical quality control procedures were applied to the set of predictors retained after univariate screening. These steps aimed to eliminate variables that could compromise the reliability of the multivariate Cox model:

1. **Low variance filtering:** First, dummy variables were assessed for low variance both *across the entire dataset (overall)* and *within subgroups stratified by event status* (i.e., diabetes onset vs. no onset). Variables exhibiting insufficient variance in either context were excluded, as such sparse features can inflate standard errors, produce unstable coefficient estimates, and reduce model robustness. This variance filtering acted as a safeguard against including statistically weak or non-informative features.
2. **Multicollinearity assessment:** Second, multicollinearity was addressed using the Variance Inflation Factor (VIF) [62], which quantifies how much the variance of a regression coefficient is inflated due to linear relationships among predictors. The VIF for a predictor $X_{j}$ is defined by Equation 2:
  $$\text{VIF}=\frac{1}{1-R_{j}^{2}}$$ (2)
  where $R_{j}^{2}$ is the coefficient of determination obtained by regressing $X_{j}$ on all other predictors in the model. Consistent with established guidelines, variables with VIF values exceeding 10 were considered to exhibit substantial multicollinearity and were therefore removed from the model to ensure stable estimation and interpretability of hazard ratios [62].

From the original set of 90 variables, the sequential univariate screening and quality control procedures resulted in 28 predictors that satisfied significance, variance, and multicollinearity criteria. Table 1 lists these predictors, which constitute the final screened feature set used as input for the next stage of model development.

**Table 1:** Predictors retained after univariate screening and quality control checks.

S. No	Variable
1	Age
2	BMI
3	Ethnicity
4	Tea intake
5	Bread type
6	Cereal type
7	Water intake
8	Cheese intake
9	Genetic sex
10	Cereal intake
11	Mental health
12	Sleep duration
13	Nap during day
14	Fresh fruit intake
15	Salt added to food
16	Usual walking pace
17	Loneliness isolation
18	Non-oily fish intake
19	Plays computer games
20	Long-standing illness
21	Sleeplessness insomnia
22	Processed meat intake
23	Worrier anxious feelings
24	Cooked vegetable intake
25	Vascular heart problems
26	Alcohol intake frequency
27	Vigorous activity days/Week
28	Difficulty getting up in morning

#### 7.1.2 Multivariate analysis

After identifying significant predictors through univariate analysis and addressing potential issues of multicollinearity and low variance, a multivariate Cox proportional hazards model was applied. Multivariate analysis is essential for understanding how multiple predictors collectively influence the time to the onset of T2DM. Unlike univariate analysis, which evaluates the relationship between each predictor and the outcome in isolation, the multivariate approach estimates the independent effect of each predictor while controlling for the influence of all other variables in the model.

The 28 features identified in univariate analysis were included in the initial multivariate model to assess their combined impact on T2DM onset. However, some variables lost their statistical significance in the multivariate context due to confounding or shared variance with other predictors. This adjustment reflects the model’s ability to isolate the unique contribution of each variable while accounting for correlations among predictors.

To refine the model, an iterative process was employed, sequentially removing non-significant variables and re-estimating the model until only predictors with statistically significant and independent associations remained. This procedure ensured that the final model retained only those features that robustly contributed to T2DM risk. Through this process, the feature set was reduced from 28 to 14 predictors.

The coefficients $\exp(\beta_{i})$ from the multivariate Cox model represent adjusted hazard ratios, quantifying the relative risk of T2DM onset associated with each predictor while holding other factors constant. For example, a dietary factor with a hazard ratio greater than 1 indicates an increased risk of T2DM with higher intake, independent of other lifestyle or demographic variables.

This stepwise refinement improves both the interpretability and reliability of the model, allowing for a focused interpretation of key predictors that significantly influence T2DM development.

#### 7.1.3 Proportional hazards assumption

Following multivariate modeling, the proportional hazards (PH) assumption was evaluated to ensure the validity of the Cox regression framework. This assumption requires that the effect of each covariate on the hazard remains constant over time, implying that hazard ratios are time-invariant. For example, if higher physical activity reduces diabetes risk by 30% (hazard ratio = 0.70), this relative effect is expected to persist throughout the follow-up period.

The PH assumption was assessed using diagnostic methods based on Schoenfeld residuals [63]. Violations can bias hazard ratio estimates; therefore, corrective strategies were applied when necessary. Two approaches were implemented: (i) stratification, allowing the baseline hazard to vary across covariate strata, and (ii) incorporation of time-dependent covariates to capture changing effects over time. In this study, non-proportionality was detected for cooked vegetable intake and long-standing illness, both of which were modeled with covariate–time interactions to preserve validity and interpretability.

These adjustments enabled the model to accommodate dynamic predictor effects, thereby enhancing explanatory accuracy while preserving the validity of the PH assumption.

#### 7.1.4 Internal validation

Model performance was evaluated using 10-fold stratified cross-validation, training on nine folds and testing on the tenth [64]. Stratification preserved the distribution of diabetes onset and censored cases across folds. L2 regularization reduced overfitting by penalizing large coefficients [65]. Performance was measured with the concordance index (C-index) [66], yielding a mean of 0.90 (SD = 0.004), indicating excellent accuracy and consistency. Low variability in performance across folds indicates that results are robust to different training and testing splits. The model was then refitted on the full dataset to maximize information, and the proportional hazards assumption was re-verified, confirming the stability and reliability of the final model.

## 8 Results and interpretation of significant predictors

This section presents the findings from the multivariate Cox proportional hazards model and discusses their implications for disease prediction, ethnic disparities, and behavioral intervention within the digital twin simulation framework.

The multivariate Cox model identified a set of psychosocial, behavioral, dietary, and demographic factors that significantly influence T2DM onset. Table 2 summarizes the selected predictors along with their coefficients (β), hazard ratios (exp(β)), percentage change in hazard (HR%), confidence intervals, and p-values. HR% was calculated as (exp⁡(β)−1)×100, and values are rounded consistently. These findings underscore the importance of modifiable non-clinical determinants alongside demographic and ethnic disparities, providing critical inputs for the digital twin simulation framework.

**Table 2:** Final selected variables and corresponding hazard ratios for T2DM risk estimated from the penalized Cox proportional hazards model. β denotes the estimated regression coefficient, exp(β) represents the hazard ratio (HR), HR(%) indicates the percentage change in hazard relative to the reference category, p denotes the p-value, and 95% confidence intervals (CI) are reported for exp(β).

Risk factors	β	exp(β)	HR(%)	p-value	95% CI for exp(β) (Lower Upper)	
Processed meat intake	0.05	1.05	5.00	p<0.01	1.02	1.07
Salt added to food	0.08	1.08	8.00	p<0.01	1.05	1.11
Cheese intake	−0.07	0.93	−7.0	p<0.01	0.91	0.95
Sugary cereals	0.15	1.17	17.00	p<0.01	1.11	1.23
Sleeplessness insomnia	0.12	1.12	12.00	p<0.01	1.07	1.19
Loneliness isolation	0.22	1.24	24.00	p<0.01	1.17	1.32
Mental health	0.26	1.29	29.00	p<0.01	1.21	1.39
Plays computer games	0.05	1.05	5.00	0.02	1.01	1.10
Difficulty getting up in morning	0.04	1.04	4.00	0.02	1.01	1.07
Ethnicity_Chinese	0.51	1.66	66.00	0.04	1.03	2.66
Ethnicity_Other ethnic group	0.62	1.86	86.00	p<0.01	1.30	2.68
Ethnicity_WB Caribbean_WB African	−0.21	0.81	−19.00	0.59	0.38	1.73
Ethnicity_White and Asian	0.10	1.10	10.00	0.75	0.60	2.02
Ethnicity_Any other mixed background	0.37	1.45	45.00	0.15	0.87	2.43
Ethnicity_Indian	0.67	1.95	95.00	p<0.01	1.40	2.71
Ethnicity_Pakistani	0.62	1.86	86.00	p<0.01	1.25	2.75
Ethnicity_Bangladeshi	0.94	2.55	155.00	0.01	1.24	5.25
Ethnicity_Any other Asian background	0.40	1.50	50.00	0.06	0.98	2.29
Ethnicity_Caribbean	0.46	1.59	59.00	0.01	1.13	2.24
Ethnicity_African	0.54	1.71	71.00	p<0.01	1.19	2.44
Age_[50–59]	−0.07	0.93	−7.00	0.45	0.77	1.12
Age_[60–70]	0.53	1.69	69.00	p<0.01	1.40	2.04
BMI_[25–29.9]_Overweight	0.11	1.11	11.00	0.27	0.92	1.34
BMI_[30–34.9]_Obesity class I	0.56	1.75	75.00	p<0.01	1.45	2.11
BMI_[35–39.9]_Obesity class II	0.76	2.14	114.00	p<0.01	1.77	2.60

WB refers to “White and Black” mixed ethnicity. “Sugary Cereals” is a binary variable where 1 indicates consumption of sugary or processed cereals Other (e.g., Cornflakes, Frosties). “Mental health” refers to individuals who have experienced anxiety or depression and have consulted a doctor for it. For “Age”, the reference category is “40–49 years”; and for “BMI” (body mass index, kg/m^2^), the reference category is “18.5–24.9” (normal weight).

To facilitate clear interpretation of regression coefficients, all predictors were coded in alignment with their original UK Biobank definitions. Categorical variables were converted into binary or dummy variables as appropriate, allowing for consistent and interpretable estimation within the Cox proportional hazards model. Psychosocial, sleep, and behavioral factors, such as loneliness, difficulty waking in the morning, and insomnia, were defined as binary exposure variables.

Salt added to food, cheese intake, processed meat intake, and plays computer games were modeled as ordinal predictors to capture graded, dose–response associations with T2DM risk. For salt added to food, category-specific effects were similar and often non-significant when modeled separately, so the variable was retained as a single ordinal term. Processed meat intake, cheese intake, and plays computer games showed a monotonic increase in hazard across ordered frequency categories and was therefore also included as an ordinal variable.

“Sugary cereals” was defined as a binary variable, with a value of 1 indicating consumption of sugary or processed breakfast cereals (e.g., Cornflakes, Frosties). “Mental health” was also modeled as a binary variable, indicating whether an individual reported a history of anxiety or depression and had consulted a medical professional for these conditions.

BMI was categorized according to World Health Organization criteria [67]. Participants classified as underweight (BMI <18.5 kg/m^2^) were excluded because of small numbers and unstable estimates. The normal-weight category (BMI 18.5–24.9 kg/m^2^) was used as the reference group in all BMI analyses. Age was specified using UK Biobank–defined decile groups (40–49, 50–59, and 60–70 years), with ages 40–49 years serving as the reference category. Modeling age categorically permits flexible estimation of age-related risk without assuming a linear relationship. Ethnicity was included as a categorical variable using dummy coding, with White ethnicity specified as the reference group.

### 8.1 Psychosocial and behavioral factors

Several psychosocial and lifestyle related variables were independently associated with the hazard of developing T2DM. Loneliness and social isolation were associated with a 24% higher hazard (HR = 1.24, 95% CI: 1.17–1.32, p<0.01), while insomnia was associated with a 12% increase in hazard (HR = 1.12, 95% CI: 1.07–1.19, p<0.01). Individuals who had consulted a doctor for anxiety or depression experienced a 29% higher hazard of T2DM onset (HR = 1.29, 95% CI: 1.21–1.39, p<0.01). Modest but significant associations were also observed for leisure behaviors. Playing computer games (HR = 1.05, 95% CI: 1.01–1.10, p=0.02) and difficulty getting up in the morning (HR = 1.04, 95% CI: 1.01–1.07, p=0.02) both elevated risk, potentially reflecting sedentary patterns, circadian misalignment, or early markers of metabolic dysfunction. Collectively, these findings underscore that psychosocial distress, disrupted sleep, and subtle lifestyle rhythms are not only quality of life issues but also measurable biological risk factors. Incorporating these predictors into digital twin models allows simulation of mental health and behavior driven trajectories, supporting the design of personalized preventive interventions.

### 8.2 Dietary habits

Several dietary variables were independently associated with the hazard of T2DM onset. Higher processed meat intake (HR = 1.05, 95% CI: 1.02–1.07, p<0.01), adding salt to food (HR = 1.08, 95% CI: 1.05–1.11, p<0.01), and consumption of sugary cereals (HR = 1.17, 95% CI: 1.11–1.23, p<0.01) were each associated with an increased hazard of developing T2DM, consistent with proposed mechanisms involving inflammation, insulin resistance, and gut microbiome disruption. Interestingly, cheese intake demonstrated a statistically significant inverse association with the hazard of T2DM onset (HR = 0.93, 95% CI: 0.91–0.95, p<0.01). While this finding contrasts with conventional dietary guidance that often cautions against high fat dairy consumption, emerging evidence indicates that certain dairy products may exert protective metabolic effects through improvements in insulin sensitivity and reductions in systemic inflammation [68–70]. One plausible biological mechanism involves the presence of vitamin K, particularly vitamin K2, in cheese. Vitamin K2 has been associated with enhanced insulin sensitivity and a lower risk of T2DM, potentially mediated through its role in osteocalcin metabolism and downstream glucose regulation [71]. Within the digital twin framework, these dietary factors constitute modifiable causal nodes and represent high leverage targets for simulating dietary intervention scenarios.

### 8.3 Age and BMI

Compared with adults aged 40–49 years, individuals aged 60–70 years exhibited a substantially higher hazard of T2DM onset (HR = 1.69, 95% CI: 1.40–2.04, p<0.01), whereas those aged 50–59 years did not differ significantly from the reference group (HR = 0.93, 95% CI: 0.77–1.12, p=0.45). Body mass index showed a clear dose-response relationship when classified according to World Health Organization categories [67]. Overweight status was not significantly associated with the hazard of T2DM (HR = 1.11, 95% CI: 0.92–1.34, p=0.27), while obesity class I (HR = 1.75, 95% CI: 1.45–2.11, p<0.01) and obesity class II (HR = 2.14, 95% CI: 1.77–2.60, p<0.01) were associated with progressively higher hazards of disease onset. These associations reaffirm excess weight and later life as critical accelerators of T2DM risk, consistent with evidence linking obesity to insulin resistance, systemic inflammation, and beta-cell dysfunction [72].

### 8.4 Ethnic disparities

In this analysis, individuals coded as British, Irish, or any other White background were grouped under the category *White* and used collectively as the reference category; this grouping reflects the broader ethnicity categorisation used in UK Biobank and ensures a sufficiently large and epidemiologically comparable baseline population. All hazard ratios for ethnicity therefore represent relative risk compared with this combined White group. Using this reference category, markedly elevated hazard ratios were observed across several ethnic groups:

- Bangladeshi participants exhibited the highest hazard of T2DM onset (HR ≈ 2.55), indicating more than a twofold increase relative to the White reference group.
- Indian, Pakistani, and Chinese participants also demonstrated substantially elevated hazards (HRs ≈ 1.95, 1.86, and 1.66, respectively).
- African and Caribbean participants experienced similarly increased hazards (HRs ≈ 1.71 and 1.59).
- Participants categorised as “Other Asian” or “Other ethnic group” showed comparable elevations in hazard relative to the reference group.
- Individuals of mixed ethnicity, including “White and Asian” (HR ≈ 1.10) and “Any other mixed background” (HR ≈ 1.45), exhibited moderately elevated hazards.

These findings are consistent with evidence from the Southall And Brent REvisited (SABRE) study, which reported a 19-year T2DM incidence of 34% among South Asians and 29% among African Caribbeans, compared to 14% among White Europeans [73, 74]. The SABRE study also noted that South Asian individuals developed T2DM at lower BMI levels, accompanied by higher truncal adiposity and insulin resistance, despite having less visceral fat. The results of this study corroborate these trends, particularly in Bangladeshi, Indian, and Pakistani populations. Further support comes from Public Health England, which highlights the increased risk and earlier onset of T2DM among individuals of South Asian, African, and Caribbean descent, often occurring at lower BMI thresholds [75]. These disparities underscore the importance of ethnically adapted screening protocols and culturally informed lifestyle interventions.

Additionally, the elevated hazards observed among mixed ethnicity groups point to a complex interplay of genetic, behavioral, and environmental determinants. This highlights the need for more granular investigations into mixed ethnicity populations, which are currently underrepresented in metabolic and epidemiological research [76].

**Collectively,** these predictors highlight how psychosocial stress, adverse diet, excess weight, and ethnic background converge to shape T2DM trajectories. For the digital twin framework, they provide both predictive power and actionable levers for simulated interventions.

#### 8.4.1 Risk score stratification and validation

Following development and internal validation of the multivariable Cox proportional hazards model, an individual risk score was computed for each participant as the linear predictor from the final fitted model. Regression coefficients represent covariate-specific contributions to the hazard, as shown in Equation 3:

$${\text{Risk Score}}_{i}=\sum_{\;j=1}^{\;p}\beta_{j}X_{ij}$$ (3)

where $\beta_{j}$ denotes the estimated coefficient for predictor j and $X_{ij}$ is the corresponding value for participant i. Categorical predictors were encoded using dummy variables, with reference categories defined in Table 2. The resulting score represents a log relative hazard summarizing the combined effects of behavioral, psychosocial, sleep-related, dietary, and demographic factors.

Once the risk scores were calculated, a decision tree classifier was trained to stratify individuals based on predicted risk, using the risk score as input and event occurrence as the target. This supervised binning approach identifies optimal split points to maximise outcome separation, following principles of supervised discretisations introduced by Fayyad and Irani [77]. The tree was constrained to five leaf nodes, identifying optimal thresholds that best separated individuals by outcome. These thresholds defined five discrete, non-overlapping risk groups: Very Low (−1.67, 0.22), Low (0.23, 0.40), Moderate (0.41, 0.70), High (0.71, 1.11), and Very High (1.12, 2.75).

To illustrate how individual predictors contribute to risk stratification, two examples are provided. An individual reporting loneliness or social isolation (X=1) and insomnia (X=1), with all other predictors at their reference levels, has a linear predictor of 0.22+0.12=0.34, which corresponds to the *Low* risk group. In contrast, an individual reporting loneliness, insomnia, regular consumption of sugary cereals, belonging to the 60–70 year age group, and obesity class I, with all remaining predictors at their reference levels, has a linear predictor of 0.22+0.12+0.15+0.53+0.56=1.58, placing them in the *Very High* risk group. These examples illustrate how specific combinations of predictors contribute additively to the overall risk score and determine risk group membership.

The discriminative performance of the resulting risk stratification was evaluated using survival analysis. Kaplan–Meier curves [78] were used to estimate diabetes-free survival across risk groups, while pairwise log-rank tests [79] assessed statistical differences between survival distributions. The Kaplan–Meier estimator accounts for censoring by incorporating information up to each participant’s last observed time point, enabling unbiased estimation of cumulative survival.

**Figure 5:**
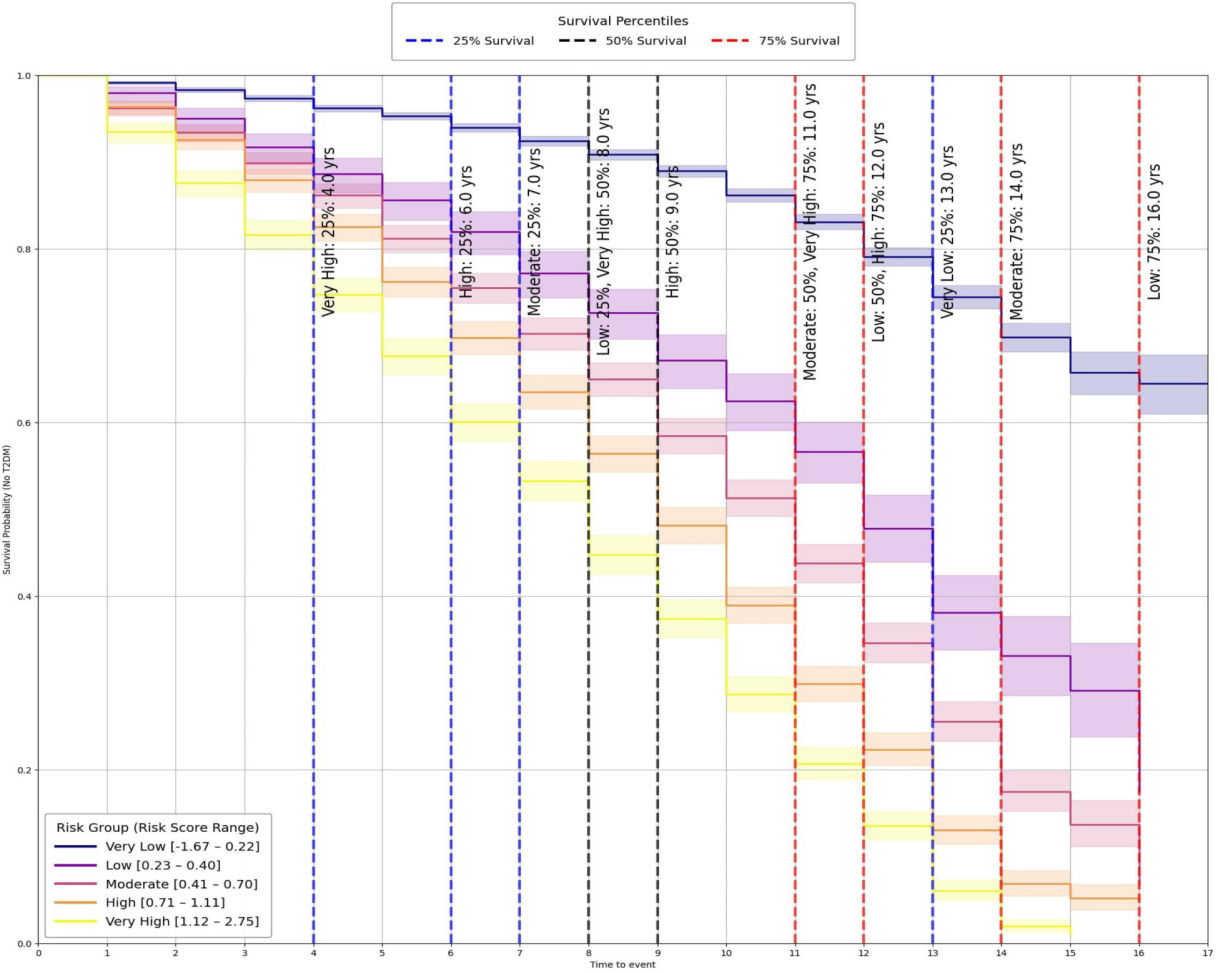
Survival probability is plotted across time for five strata: very low, low, moderate, high, and very high (defined by risk score ranges). Shaded bands indicate confidence intervals. Vertical dashed lines mark 25%, 50%, and 75% survival percentiles, with labels showing estimated survival times for each group. The curves demonstrate clear risk separation, with higher risk groups experiencing earlier survival decline.

As shown in Figure 5, the resulting curves demonstrated clear and progressive separation across strata, with higher risk groups exhibiting earlier and steeper declines in diabetes free survival. Vertical dashed lines indicate the estimated 25%, 50%, and 75% survival percentiles for each group, illustrating systematic shifts toward shorter survival times with increasing risk. Pairwise log rank tests further confirmed that differences between groups were statistically significant (p<0.01, Table 3a), demonstrating that the stratification captures both major and incremental variation in diabetes onset risk. To provide interpretable time-based summaries, survival percentiles were also computed (Table 3b), revealing a monotonic shift toward earlier diabetes onset with increasing risk category. For instance, individuals in the *Very High* group reached 25% incidence by year 4 and 75% by year 11, whereas the *Very Low* group crossed the 25% threshold only after 13 years and did not reach higher incidence levels during follow-up. For the very low risk group, the median (50%) and 75% survival times were not observed during follow up and are therefore reported as ∞, indicating that fewer than 50% and 75% of participants experienced the event within the observation window.

**Table 3:** Panel (a) reports pairwise log-rank test outcomes: *Comparison* shows the two groups tested, *Test Statistic* is the log-rank $\chi^{2}$ value, and p-value indicates significance. Panel (b) shows survival time percentiles for each group, with *Score Range* defining the risk category and 25%, 50% (median), and 75% giving survival times in years. “∞” denotes that survival did not fall below the percentile threshold during follow-up.

(a) Pairwise log-rank test results				
Comparison	Test statistic		p-value	
Very low vs. very high	5,255.49		p<0.01	
Very low vs. high	3,662.31		p<0.01	
Very low vs. moderate	2,072.38		p<0.01	
Low vs. very high	409.51		p<0.01	
Moderate vs. very high	353.93		p<0.01	
Very low vs. low	517.59		p<0.01	
Low vs. high	189.67		p<0.01	
Moderate vs. high	95.38		p<0.01	
High vs. very high	89.86		p<0.01	
Low vs. moderate	46.71		p<0.01	

(b) Survival time percentiles by risk group				
Risk group	Score range	25%	50%	75%
Very Low	[−1.67, 0.22]	13.00	∞	∞
Low	[0.23, 0.40]	8.00	12.00	16.00
Moderate	[0.41, 0.70]	7.00	11.00	14.00
High	[0.71, 1.11]	6.00	9.00	12.00
Very High	[1.12, 2.75]	4.00	8.00	11.00

**Taken together,** this stratification highlights the practical utility of the digital twin framework beyond statistical validation. By translating continuous outputs from the Cox model into discrete, time-sensitive categories, the system provides thresholds that can inform clinical decision-making and public health planning. These findings show that psychosocial and behavioral risk factors can be structured into groups that are both statistically distinct and clinically meaningful, reinforcing the value of this framework for individualized prevention and population-level screening.

## 9 Causal inference and simulation of intervention effects

While Cox models identify statistical associations, they do not establish whether modifying risk factors changes outcomes. This limitation is crucial for a DT, where simulating counterfactual scenarios (e.g., “What if loneliness were reduced?”) is essential. To address this, a causal inference framework was integrated, enabling estimation of intervention effects on T2DM progression. By quantifying the impact of modifiable variables such as sleep, diet, and psychosocial stress, the DT supports modeling of how risk may be reduced rather than only predicting who is at risk.

**Figure 6:**
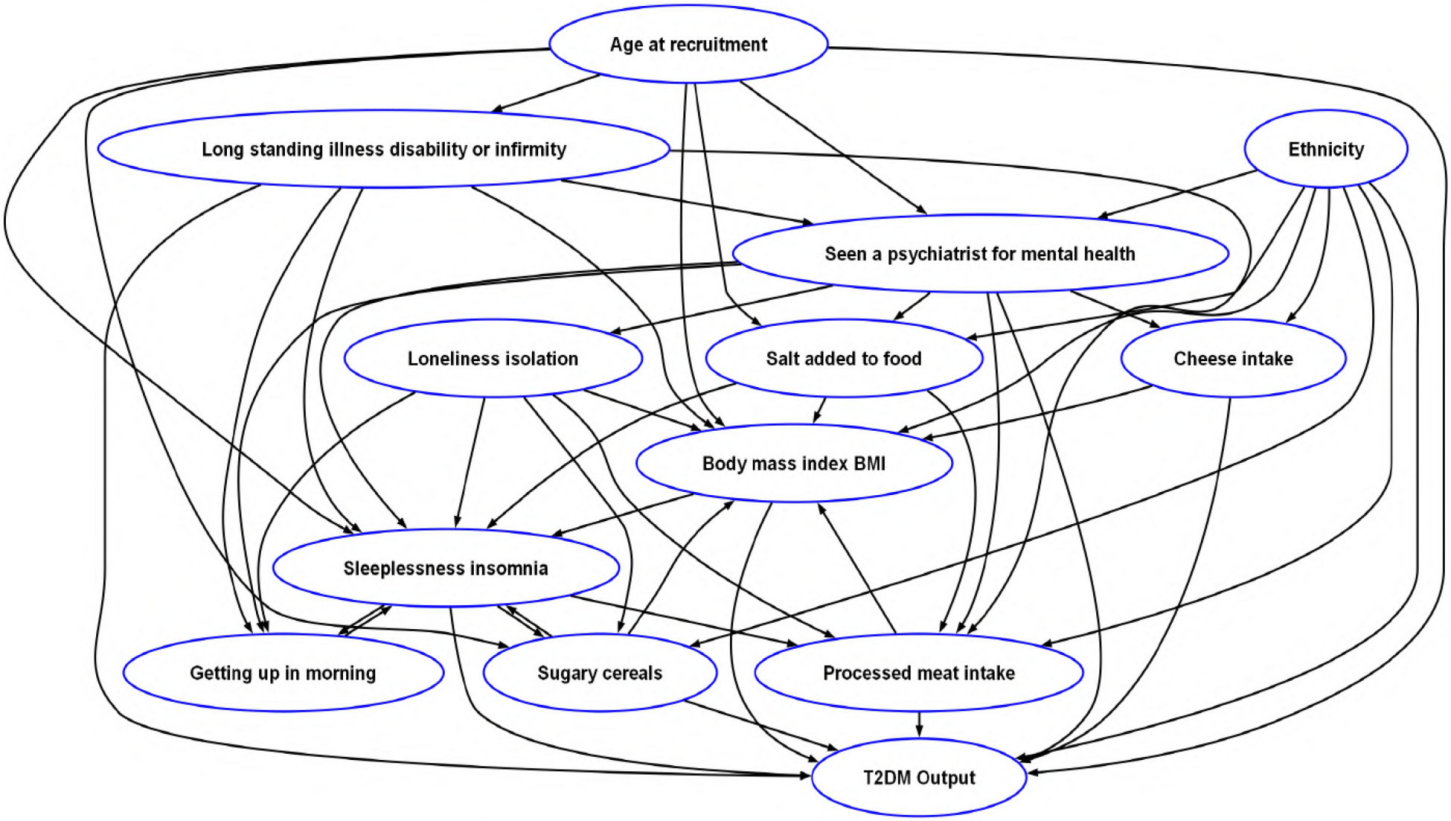
Expert-derived causal graph encoding prior knowledge on T2DM progression, consisting of 13 nodes and 49 directed edges denoting hypothesized causal relationships among key lifestyle, demographic, dietary, psychosocial and behavioral factors.

The framework proceeded in two stages. First, a domain informed Directed Acyclic Graph (DAG) was constructed to encode expert knowledge about hypothesized causal pathways between variables. (Figure 6). Second, causal effects were estimated via propensity score matching, backdoor adjustment, and robustness checks. This approach shifts the DT from correlation-based prediction to intervention-oriented simulation.

### 9.1 Stage 1: construction of DAG

To ensure transparency in the causal modeling, a detailed rationale is provided for the relationships embedded in the expert-driven DAG (Figure 6). Each directed edge in the graph reflects prior knowledge derived from epidemiological and clinical literature, linking psychosocial, dietary, behavioral, and demographic factors to the risk of developing T2DM. The following section presents the justification for each major domain included in the DAG.

#### 9.1.1 Age, BMI, and metabolic pathways

Age was modeled as an upstream determinant shaping BMI, sleep, diet, long-standing illness, and T2DM, reflecting its role in multimorbidity, sleep disruption, and dietary shifts [80, 81]. BMI was positioned centrally as a mediator between psychosocial and dietary inputs and diabetes risk. It is influenced by diet and stress, interacts bidirectionally with sleep, and, when elevated, drives insulin resistance through impaired glucose regulation [82–84].

#### 9.1.2 Dietary influences

Dietary habits were modeled as influencing both BMI and T2DM directly. High intake of sugary or processed cereals is associated with weight gain and increased T2DM risk [85]. In contrast, cheese intake may be protective due to its protein and fat content, which can enhance satiety and insulin sensitivity [69, 86], and potentially through its vitamin K2 content [71]. Processed meats are linked to both elevated BMI and direct metabolic disruption via pro-inflammatory effects [87]. Salt addition was included due to evidence that it may promote weight gain through mechanisms such as fluid retention and altered taste perception leading to higher energy intake [88].

#### 9.1.3 Psychosocial and sleep-related factors

Loneliness and social isolation were modeled as upstream drivers influencing diet, sleep, and BMI [89]. Psychiatric history was linked to disordered eating, poor sleep, elevated BMI, and increased T2DM risk [90]. Sleep disturbances were incorporated as both causes and consequences of metabolic dysfunction: insomnia affects cortisol, appetite, and glucose metabolism, while difficulty waking was treated as a proxy for circadian disruption [91, 92].

#### 9.1.4 Ethnicity and physical health conditions

Ethnicity was modeled as a root variable shaping dietary patterns, BMI, mental health, and T2DM risk, reflecting disparities in prevalence, cultural stressors, and lifestyle determinants [93–95]. Long-standing illness was included as a driver of mental health problems, disrupted sleep, altered diet, elevated BMI, and direct metabolic vulnerability, consistent with evidence on multimorbidity and heightened T2DM risk [96–98].

Together, these justifications ensured the DAG reflected plausible causal pathways, forming the foundation for intervention simulation.

### 9.2 Stage 2: causal inference and simulation of intervention effects

To move beyond association and simulate actionable interventions, causal inference methods within the potential outcomes framework were applied. The Average Treatment Effect on the Treated (ATT) [99] was estimated for key modifiable factors such as BMI and psychosocial stressors. Treatments were operationalised as binary exposures based on clinically meaningful thresholds (e.g., obesity defined as BMI ≥ 30 kg/m^2^, and psychosocial stressors defined as presence or absence of loneliness, insomnia, or mental health consultation). This approach was chosen to approximate the effects of real-world interventions in an observational dataset, where randomized trials are not feasible. This stage involved three methodological steps: propensity score matching, regression-based backdoor adjustment, and robustness checks.

#### 9.2.1 Propensity score matching and ATT

The ATT estimation measures the causal effect of a treatment or condition (e.g., having obesity) on those who actually experienced it. Since this is an observational dataset, confounding variables may bias naive comparisons. To address this, a 2-to-1 propensity score matching algorithm was used, pairing each treated individual with two controls with similar covariate profiles. A propensity score reflects the conditional probability of receiving the treatment given observed covariates. Matching individuals with similar scores balances the distribution of confounding variables between treated and control groups. This mitigates selection bias and allows for fairer estimation of causal effects [100].

#### 9.2.2 Standardized mean differences

To ensure the quality of covariate balance, *Standardised Mean Differences* (SMDs) were computed before and after weighting for each confounder [101]. This diagnostic helps verify that the re-weighted treatment and control groups are statistically comparable on the observed covariates, which is a prerequisite for valid causal inference.

#### 9.2.3 Backdoor adjustment and regression modeling

Once matching was performed and covariate balance confirmed, the ATT was estimated using DoWhy’s regression-based backdoor adjustment [30]. By conditioning on a suitable set of covariates, the backdoor criterion ensures that non-causal pathways between the treatment and outcome are blocked, allowing identification of the causal effect.

Regression was then used as the estimation procedure to implement this backdoor adjustment on the matched dataset, further reducing residual imbalance and improving statistical precision. This combined approach, matching followed by regression-based backdoor adjustment, reduces model dependence and enhances robustness. The resulting ATT estimates reflect the isolated causal impact of treatment on the outcome among the treated population.

#### 9.2.4 Robustness checks

To evaluate the reliability and validity of the estimated causal effects, several robustness tests were conducted as recommended by Sharma et al. [31]:

- **Placebo treatment tests:** Treatment labels were randomly reassigned, and causal effects were re-estimated. As expected, these placebo treatments produced near-zero effects with non-significant p-values (p>0.05), confirming that the original effects presented in this study were not due to random correlations.
- **Subset sampling tests:** The dataset was randomly split into multiple subsets, and causal estimates were recalculated for each. Results showed minimal deviation (<1%) from the original ATT, indicating that the results are not sensitive to specific subgroups.
- **Bootstrap refutation tests:** By resampling the data with replacement and recalculating the ATT, empirical distributions of treatment effects were generated. The original estimates fell within the bootstrap confidence intervals, indicating strong internal consistency.
- **Hidden confounding/sensitivity analysis:** Simulated unobserved confounders were added to assess the vulnerability of causal effects to omitted variable bias. The ATT estimates remained stable within acceptable ranges, confirming robustness to moderate unmeasured confounding.

Taken together, these refutation strategies strengthen the credibility of the causal estimates by demonstrating that results are not driven by model specification or sampling variability. This process ensures that the estimated causal effects are credible, allowing digital twin simulations to move beyond identifying “who is at risk” toward modeling “how risk can be reduced.” These considerations provide a robust methodological basis for applying causal inference within digital twin simulations to evaluate hypothetical interventions.

##### 9.2.4.1 Example of the causal estimation workflow

Consider estimation of the causal effect of obesity (BMI ≥ 30 kg/m^2^) on the risk of T2DM. Individuals classified as obese formed the treated group, while non-obese individuals served as potential controls. Propensity score matching was first applied to pair each obese individual with two non-obese individuals (2-to-1 propensity score matching) who had similar values of age, ethnicity, long-standing illness, and relevant dietary indicators. Covariate balance between the treated and matched control groups was then assessed using SMD, and only matched samples achieving acceptable balance were retained for causal estimation.

After covariate balance was confirmed, regression-based backdoor adjustment was applied to the matched dataset, conditioning on the covariates identified by the causal DAG to block all backdoor paths between obesity and T2DM. The resulting ATT represents the estimated difference in T2DM risk that obese individuals would experience under a counterfactual scenario in which they were not obese, holding observed confounders constant. This estimate was then subjected to the refutation and sensitivity analyses described above to evaluate the robustness of the causal effect.

## 10 Results and discussion from causal modeling

To enhance interpretability and clinical relevance, causal modeling was organized around three research questions linking BMI, psychosocial stressors (insomnia, loneliness, mental health), and dietary behaviors to T2DM risk. These questions form the analytical basis of the DT prototype, enabling simulation of individualized “what-if” scenarios where modifiable psychosocial and behavioral factors are altered.

- **Q1:** How does BMI (across normal weight, overweight, and obesity) influence psychosocial stressors, and to what extent do these stressors mediate the overall risk of developing T2DM?
- **Q2:** How do individual psychosocial stressors such as insomnia, loneliness, and poor mental health separately influence dietary behavior and consequently the risk of T2DM?
- **Q3:** How do these psychosocial stressors, when occurring together, jointly influence dietary behavior and the downstream risk of T2DM?
- **Q4:** What is the direct effect of simultaneously improving psychosocial stressors on the risk of developing T2DM?

These questions not only shaped the causal modeling process but also form the decision logic underlying the DT prototype. By quantifying both direct and indirect pathways, particularly those mediated by modifiable psychosocial and behavioral factors, this stage allows the DT to simulate realistic and individualized intervention outcomes.

Importantly, this causal analysis builds upon the predictive foundation established in Stage 1, which used multivariate Cox modeling to identify baseline predictors of T2DM onset. Stage 2 advances from prediction to simulation by applying formal causal inference methods (e.g., propensity score matching and backdoor regression adjustment) to estimate counterfactual effects, with effects reported on the probability scale where indicated (ATT). All findings were rigorously validated using refutation strategies, including placebo testing, subset evaluation, bootstrap resampling, and sensitivity analyses. Confounders for all paths were selected *a priori* from the DAG (age, ethnicity, long-term condition, and diet indicators where relevant). Diet variables were treated as pre-exposure preferences to reduce residual confounding; outcome models for diet mediators also adjusted for the exposure and other diet indicators.

### 10.1 Q1: effects of BMI on psychosocial stressors and T2DM mediation

This analysis examines how BMI categories interact with psychosocial stressors to shape diabetes risk within the study cohort. Individuals were classified as normal weight (18.5–24.9 kg/m^2^), overweight (25.0–29.9 kg/m^2^), or obese (≥30 kg/m^2^), with each category coded as binary (1 = within range, 0 = outside range). Since the dataset does not include underweight individuals (BMI < 18.5 kg/m^2^), these binary comparisons focus exclusively on normal weight, overweight, and obesity. Stability of the estimated effects within this cohort was supported by robustness checks, strengthening confidence in their internal consistency.

Causal mediation analysis suggests that stress-related factors, particularly insomnia, loneliness, and poor mental health, partly explain the association between BMI and T2DM risk. Effects are estimated on the probability scale using ATT; the direct effect is a controlled direct effect (BMI’s impact on T2DM when the mediator is held fixed), and indirect effects are computed as $E_{1}\times E_{2}$ (predictor–mediator effect multiplied by mediator–outcome effect). The total effect is the sum of direct and indirect components.

#### 10.1.1 Direct effects of BMI on T2DM risk

As shown in Table 4, in this analysis BMI exhibited strong and statistically robust controlled direct effects on T2DM risk. Normal weight was protective, ATT =−0.244 (95% CI: −0.2568 to −0.2332), corresponding to a 24.4 percentage-point lower absolute risk. Overweight increased risk, ATT = 0.1229 (95% CI: 0.1114 to 0.1333), a 12.3 percentage-point higher risk, while obesity further elevated risk, ATT = 0.3493 (95% CI: 0.3376 to 0.3608), a 34.9 percentage-point higher risk. These direct effects accounted for approximately 95–99% of the total estimated effect, indicating that BMI explains most of the modeled association with T2DM risk. Psychosocial mediators contributed only a small fraction on the absolute scale, though still detectable and meaningful. Robustness checks supported the stability of these estimates, strengthening confidence in their interpretation as actionable insights.

**Table 4:** Estimated direct, indirect, and total causal effects of BMI on T2DM risk mediated by insomnia, loneliness, and mental health, estimated using DoWhy.

BMI	BMI → Insomnia ($E_{1}$)	Insomnia → T2DM ($E_{2}$)	BMI → T2DM (Direct/Total)
18.5–24.9	$E_{1}$: −0.0485 (CI: −0.0645, −0.0351)	$E_{2}$: 0.0743 (CI: 0.0610, 0.0876)	Direct: −0.244 (CI: −0.2568, −0.2332)
	Placebo: −0.0011 (p=0.86)	Indirect =−0.0036	Total =−0.2477
	Subset: −0.0490 (p=0.92)	Placebo: −0.0001 (p=0.98)	Placebo: 0.0002 (p=0.90)
	Bootstrap: −0.0470 (p=0.92)	Subset: 0.0741 (p=0.98)	Subset: −0.2442 (p=1.00)
	Sensitivity: −0.0485 (−0.0518, −0.0437)	Bootstrap: 0.0748 (p=0.96)	Bootstrap: −0.2445 (p=0.88)
		Sensitivity: 0.0743 (0.0821, 0.0853)	Sensitivity: −0.2441 (−0.2428, −0.0338)
25–30	$E_{1}$: 0.0254 (CI: 0.0131, 0.0370)	$E_{2}$: 0.0743 (CI: 0.0604, 0.0882)	Direct: 0.1229 (CI: 0.1114, 0.1333)
	Placebo: 0.0003 (p=0.90)	Indirect = 0.0019	Total = 0.1248
	Subset: 0.0252 (p=0.94)	Placebo: −0.0001 (p=0.96)	Placebo: −0.0001 (p=0.96)
	Bootstrap: 0.0251 (p=0.86)	Subset: 0.0746 (p=0.98)	Subset: 0.1226 (p=0.94)
	Sensitivity: 0.0254 (0.0224, 0.0254)	Bootstrap: 0.0743 (p=0.98)	Bootstrap: 0.1232 (p=0.98)
		Sensitivity: 0.0743 (−0.0744, 0.0775)	Sensitivity: 0.1229 (0.1149, 0.1258)
>30	$E_{1}$: 0.1547 (CI: 0.1409, 0.1706)	$E_{2}$: 0.0743 (CI: 0.0604, 0.0882)	Direct: 0.3493 (CI: 0.3376, 0.3608)
	Placebo: 0.0002 (p=0.94)	Indirect = 0.0115	Total = 0.3608
	Subset: 0.1548 (p=0.96)	Placebo: −0.0001 (p=0.96)	Placebo: 0.0006 (p=0.96)
	Bootstrap: 0.1540 (p=0.88)	Subset: 0.0746 (p=0.98)	Subset: 0.3490 (p=0.96)
	Sensitivity: 0.1547 (0.1541, 0.1549)	Bootstrap: 0.0743 (p=0.98)	Bootstrap: 0.3502 (p=0.80)
		Sensitivity: 0.0743 (−0.0744, 0.0775)	Sensitivity: 0.3493 (0.2947, 0.3515)

BMI	BMI → Loneliness ($E_{1}$)	Loneliness → T2DM ($E_{2}$)	BMI → T2DM (Direct/Total)
18.5–24.9	$E_{1}$: −0.0356 (CI: −0.0465, −0.0240)	$E_{2}$: 0.1109 (CI: 0.0957, 0.1262)	Direct: −0.2441 (CI: −0.2565, −0.2329)
	Placebo: −0.0008 (p=0.96)	Indirect =−0.0040	Total =−0.2481
	Subset: −0.0356 (p=0.96)	Placebo: 0.0005 (p=0.88)	Placebo: −0.0008 (p=0.94)
	Bootstrap: −0.0350 (p=0.84)	Subset: 0.1111 (p=1.00)	Subset: −0.2441 (p=0.90)
	Sensitivity: −0.0356 (−0.0358, −0.0285)	Bootstrap: 0.1111 (p=1.00)	Bootstrap: −0.2448 (p=0.98)
		Sensitivity: 0.1109 (−0.0138, 0.1357)	Sensitivity: −0.2441 (−0.2533, −0.0203)
25–30	$E_{1}$: 0.0299 (CI: 0.0193, 0.0394)	$E_{2}$: 0.1212 (CI: 0.1054, 0.1371)	Direct: 0.1200 (CI: 0.1086, 0.1300)
	Placebo: 0.0005 (p=0.96)	Indirect = 0.0036	Total = 0.1236
	Subset: 0.0298 (p=0.98)	Placebo: −0.0004 (p=0.98)	Placebo: 0.0007 (p=0.96)
	Bootstrap: 0.0311 (p=0.80)	Subset: 0.1216 (p=0.80)	Subset: 0.1200 (p=0.92)
	Sensitivity: 0.0299 (0.0300, 0.0307)	Bootstrap: 0.1214 (p=1.00)	Bootstrap: 0.1194 (p=0.94)
		Sensitivity: 0.1212 (−0.1032, 0.1383)	Sensitivity: 0.1200 (0.1208, 0.1275)
>30	$E_{1}$: 0.1183 (CI: 0.1055, 0.1299)	$E_{2}$: 0.0999 (CI: 0.0849, 0.1148)	Direct: 0.3069 (CI: 0.2939, 0.3207)
	Placebo: 0.0001 (p=0.94)	Indirect = 0.0118	Total = 0.3187
	Subset: 0.1180 (p=0.86)	Placebo: 0.0006 (p=0.90)	Placebo: 0.0014 (p=0.72)
	Bootstrap: 0.1188 (p=1.00)	Subset: 0.1001 (p=0.94)	Subset: 0.3074 (p=0.90)
	Sensitivity: 0.1183 (0.1185, 0.1191)	Bootstrap: 0.0983 (p=0.92)	Bootstrap: 0.3073 (p=0.92)
		Sensitivity: 0.0999 (−0.1053, 0.0985)	Sensitivity: 0.3069 (−0.0262, 0.3068)

BMI	BMI → Mental health ($E_{1}$)	Mental health → T2DM ($E_{2}$)	BMI → T2DM (Direct/Total)
18.5–24.9	$E_{1}$: −0.0129 (CI: −0.0230, −0.0028)	$E_{2}$: 0.1192 (CI: 0.0998, 0.1385)	Direct: −0.2462 (CI: −0.2593, −0.2332)
	Placebo: 0.0000 (p=0.88)	Indirect =−0.0015	Total =−0.2477
	Subset: −0.0127 (p=1.00)	Placebo: 0.0009 (p=0.92)	Placebo: 0.0002 (p=0.98)
	Bootstrap: −0.0124 (p=0.90)	Subset: 0.1186 (p=0.88)	Subset: −0.2457 (p=0.90)
	Sensitivity: −0.0129 (−0.0240, −0.0130)	Bootstrap: 0.1190 (p=0.98)	Bootstrap: −0.2461 (p=0.94)
		Sensitivity: 0.1192 (0.1113, 0.1207)	Sensitivity: −0.2462 (−0.2434, −0.0035)
25–30	$E_{1}$: 0.0170 (CI: 0.0102, 0.0257)	$E_{2}$: 0.1249 (CI: 0.1048, 0.1450)	Direct: 0.1208 (CI: 0.1103, 0.1313)
	Placebo: −0.0006 (p=0.78)	Indirect = 0.0021	Total = 0.1229
	Subset: 0.0177 (p=0.80)	Placebo: 0.0001 (p=0.76)	Placebo: −0.0005 (p=0.84)
	Bootstrap: 0.0184 (p=1.00)	Subset: 0.1243 (p=0.90)	Subset: 0.1210 (p=0.88)
	Sensitivity: 0.0170 (0.0173, 0.0180)	Bootstrap: 0.1231 (p=0.90)	Bootstrap: 0.1205 (p=0.86)
		Sensitivity: 0.1249 (0.1167, 0.1266)	Sensitivity: 0.1208 (0.1213, 0.1264)
>30	$E_{1}$: 0.0803 (CI: 0.0707, 0.0879)	$E_{2}$: 0.1142 (CI: 0.0953, 0.1331)	Direct: 0.3145 (CI: 0.3014, 0.3276)
	Placebo: −0.0002 (p=0.92)	Indirect = 0.0092	Total = 0.3237
	Subset: 0.0795 (p=0.90)	Placebo: −0.0001 (p=0.98)	Placebo: −0.0003 (p=0.94)
	Bootstrap: 0.0793 (p=0.92)	Subset: 0.1134 (p=0.80)	Subset: 0.3148 (p=0.88)
	Sensitivity: 0.0803 (0.0789, 0.0793)	Bootstrap: 0.1145 (p=0.96)	Bootstrap: 0.3139 (p=0.84)
		Sensitivity: 0.1142 (0.1158, 0.1238)	Sensitivity: 0.3145 (0.1610, 0.3247)

$E_{1}$ denotes the effect of BMI on the mediator, $E_{2}$ denotes the effect of the mediator on T2DM risk, and indirect effects are computed as $E_{1}\times E_{2}$. Direct effects represent controlled direct effects of BMI on T2DM, and total effects are the sum of direct and indirect components. All effects are reported on the probability scale as ATT. Confounders were selected from the DAG and include ethnicity, age, long-term condition, and dietary variables (cheese, salt, sugary cereals, processed meat) for all paths. The BMI → T2DM model additionally conditions on the mediator to obtain a controlled direct effect. Sensitivity entries report refutation diagnostics rather than conventional confidence intervals.

#### 10.1.2 Psychosocial mediation pathways: insomnia, loneliness, and mental health

Table 4 shows that indirect effects were modest but consistent on the absolute scale. For insomnia, the indirect effect was −0.36 percentage points at normal BMI, 0.19 percentage points at overweight, and 1.15 percentage points at obesity. Loneliness was the largest pathway: −0.40 percentage points at normal BMI, 0.36 percentage points at overweight, and 1.18 percentage points at obesity. Mental health contributed the smallest indirect effects: −0.15 percentage points, 0.21 percentage points, and 0.92 percentage points across normal, overweight, and obesity, respectively. These magnitudes increase with BMI, consistent with a dose–response pattern in which higher BMI is associated with amplified psychosocial and metabolic risk.

#### 10.1.3 Refutation and robustness tests

Across all BMI groups and mediators, robustness checks, including placebo, subset, bootstrap, and sensitivity analyses, consistently validated the causal estimates. Placebo effects were negligible (p-values > 0.84), subset and bootstrap tests showed near-identical estimates to primary models (differences <1%), and sensitivity analyses confirmed that indirect effects remained stable even when accounting for potential unmeasured confounding. These sensitivity entries reflect robustness checks from DoWhy refuters (placebo, subset, bootstrap, unobserved common cause), not conventional confidence intervals. These robustness tests collectively support the internal reliability of the estimated direct and indirect effects within the specified causal model.

#### 10.1.4 Discussion

1. **Psychosocial contribution:** Metabolic pathways remain the dominant driver of the BMI–T2DM relationship, yet insomnia, loneliness, and mental health difficulties consistently contributed measurable risk. Across mediators, loneliness accounted for the largest share of indirect effect, followed by insomnia and then mental health. Loneliness emerged as the strongest mediator in obesity (up to 3.7% of the total effect), consistent with evidence linking social isolation, sleep disturbance, and adverse metabolic outcomes. While secondary to metabolic drivers, psychosocial stressors represent meaningful amplifiers of estimated diabetes risk within the modeled causal framework.
2. **Dual burden of elevated BMI:** Within this study population, elevated BMI was associated with both metabolic and psychosocial burdens. Although more than 95% of the estimated risk operated through metabolic pathways, psychosocial pathways may provide complementary intervention targets, especially where sleep, loneliness, and mental health challenges co-occur with obesity.
3. **Protective role of normal BMI:** Normal BMI was associated with negative indirect effects across all three mediators. This pattern suggests not only reduced metabolic load but also reduced exposure to psychosocial stressors, indicating a form of dual protection. Maintaining a healthy weight therefore appears to buffer against both physiological and psychosocial contributors to diabetes risk, reinforcing the potential importance of integrated lifestyle interventions.
4. **Dose-response gradient:** Indirect psychosocial effects increased progressively with BMI, highlighting a dose-response gradient. These findings underscore the value of early intervention to prevent psychosocial as well as metabolic risk accumulation, subject to the assumptions of the modeled pathways.
5. **Methodological rigor:** Concordant placebo, subset, bootstrap, and sensitivity analyses confirmed the robustness of estimates, reducing the likelihood that mediation effects reflect confounding or model misspecification. This strengthens confidence that psychosocial stressors are genuine secondary pathways within the specified causal model.

#### 10.1.5 Implications for clinical practice, and public health

- **Clinical practice:** These findings suggest that prevention strategies may benefit from extending beyond metabolic risk management to incorporate psychosocial dimensions. Routine screening for sleep disturbance, loneliness, and mental health could complement BMI and metabolic assessments, particularly in overweight and obese patients.
- **Public health:** Weight management programs could embed sleep hygiene and psychosocial support to maximize preventive impact. For example, under the modeled counterfactual scenarios, the indirect effect of insomnia in obesity (0.0115) could correspond to more than 100,000 potentially preventable diabetes cases per 10 million people. Similarly, loneliness (0.0118) and poor mental health (0.0092) in obesity could each account for tens of thousands of additional preventable cases under comparable assumptions. Together, these figures highlight that psychosocial pathways, though secondary, have substantial aggregate impact.

#### 10.1.6 Implications for policymakers

For policymakers, these findings highlight several potential opportunities for consideration:

1. Consider incorporating psychosocial stress screening in national diabetes risk assessment, mandating or incentivizing the inclusion of validated tools to assess sleep disturbances, loneliness, and mental health issues within primary care and preventive health screenings.
2. Support funding for integrated intervention programs, encouraging or prioritizing multi-faceted lifestyle interventions that target both metabolic and psychosocial factors, rather than relying solely on weight loss or dietary modifications.
3. Promote intersectoral collaborations by fostering partnerships between health agencies, community organizations, and mental health services to ensure that psychosocial support structures are accessible to populations at highest risk.
4. Leverage data-driven resource allocation by using data from studies like this to identify high-risk populations and allocate resources for targeted prevention campaigns, including culturally sensitive interventions addressing both BMI and psychosocial well-being.

By prioritizing these holistic, evidence-based strategies, policymakers can promote integrated diabetes prevention efforts that recognize both the physical and psychosocial dimensions of health. Such comprehensive approaches have the potential to advance equitable public health outcomes and contribute to reducing the growing burden of diabetes, pending validation in diverse real-world settings.

### 10.2 Q2. Individual psychosocial stressors, diet, and T2DM risk

This analysis assessed insomnia, loneliness, and poor mental health as psychosocial stressors influencing T2DM risk via direct and dietary-mediated pathways. Processed meat intake was dichotomized as high if the reported frequency was ≥3 on the original scale. Cheese intake was dichotomized as high if the reported frequency was ≥4. Salt added to food was dichotomized as high if the reported frequency was ≥3. Effects are estimated on the probability scale using the Average Treatment Effect on the Treated (ATT). Direct effects are controlled direct effects (conditioning on the dietary mediator), indirect effects are computed as $E_{1}\times E_{2}$ (exposure → mediator effect multiplied by mediator → outcome effect), and total effects are the sum of direct and indirect components. All mediators are binary; effects are interpreted as percentage-point changes in absolute risk. In this study, positive indirect effects indicate higher T2DM risk transmitted through the mediator. Negative indirect effects indicate risk reduction through the mediator within the modeled counterfactual framework. Importantly, when the exposure reduces a protective food and that food is associated with lower T2DM risk, the indirect effect will be positive, reflecting higher diabetes risk despite lower consumption of the protective food.

As summarized in Tables 5, 6, direct effects were large and broadly similar across models, with minor variation because the controlled direct effect is estimated within each mediator model. For *insomnia*, the direct ATT ranged from 0.306 to 0.366 across mediators. For *loneliness*, the direct ATT clustered tightly around 0.363–0.365. For *mental health*, it was 0.349–0.350. In short, within this study population and under the modeled assumptions, each stressor was associated with an estimated increase in absolute T2DM risk of approximately 35 percentage points across models, regardless of the diet mediator chosen.

**Table 5:** Impact of insomnia and loneliness on dietary behavior and T2DM risk: estimates and robustness checks. $E_{1}$ denotes the effect of the psychosocial exposure on the dietary mediator, $E_{2}$ denotes the effect of the mediator on T2DM risk, and indirect effects are computed as $E_{1}\times E_{2}$. Direct effects represent controlled direct effects of the exposure on T2DM, and total effects are the sum of direct and indirect components. All effects are reported on the probability scale as ATT. Confounders were selected from the DAG: age, long-term condition, and ethnicity. For psychosocial exposures, the other two stressors were included to block shared causes. In $E_{2}$ models, the exposure and non-focal diet indicators were added to reduce residual confounding. Direct-effect models conditioned on the mediator to estimate controlled direct effects. Refuters served as robustness checks rather than conventional confidence intervals. Sensitivity entries report refutation diagnostics rather than conventional confidence intervals.

Mediator	Insomnia → Mediator ($E_{1}$)	Mediator → T2DM ($E_{2}$)	Insomnia → T2DM (Direct/Total)
Processed meat	$E_{1}$: 0.0369	$E_{2}$: 0.1083	Direct: 0.3661
	(CI: 0.0197, 0.0541)	(CI: 0.0948, 0.1218)	(CI: 0.3526, 0.3795)
	Placebo: −0.0010 (p=0.84)	Indirect = 0.0040	Total = 0.3661
	Subset: 0.0365 (p=0.90)	Placebo: 0.0006 (p=0.94)	Placebo: −0.0001 (p=0.98)
	Bootstrap: 0.0370 (p=0.94)	Subset: 0.1081 (p=0.92)	Subset: 0.3663 (p=0.98)
	Sensitivity: 0.0369 (0.0368, 0.0376)	Bootstrap: 0.1081 (p=0.92)	Bootstrap: 0.3652 (p=0.92)
		Sensitivity: 0.1083 (0.1058, 0.1098)	Sensitivity: 0.3661 (0.3664, 0.3715)
Salt added	$E_{1}$: 0.0661	$E_{2}$: 0.0753	Direct: 0.3061
	(CI: 0.0539, 0.0784)	(CI: 0.0557, 0.0937)	(CI: 0.2923, 0.3199)
	Placebo: 0.0007 (p=0.86)	Indirect = 0.0050	Total = 0.3111
	Subset: 0.0657 (p=0.90)	Placebo: −0.0001 (p=0.98)	Placebo: 0.0005 (p=0.98)
	Bootstrap: 0.0662 (p=0.92)	Subset: 0.0750 (p=0.96)	Subset: 0.3064 (p=0.96)
	Sensitivity: 0.0661 (0.0653, 0.0658)	Bootstrap: 0.0763 (p=1.00)	Bootstrap: 0.3067 (p=0.98)
		Sensitivity: 0.0754 (0.0638, 0.0733)	Sensitivity: 0.3061 (0.3049, 0.3081)
Sugary cereals	$E_{1}$: 0.0307	$E_{2}$: 0.0856	Direct: 0.3661
	(CI: 0.0181, 0.0432)	(CI: 0.0673, 0.1033)	(CI: 0.3526, 0.3795)
	Placebo: −0.0007 (p=0.86)	Indirect = 0.0026	Total = 0.3687
	Subset: 0.0303 (p=0.82)	Placebo: 0.0002 (p=1.00)	Placebo: −0.0011 (p=0.84)
	Bootstrap: 0.0307 (p=0.96)	Subset: 0.0862 (p=0.76)	Subset: 0.3660 (p=0.94)
	Sensitivity: 0.0307 (0.0308, 0.0311)	Bootstrap: 0.0852 (p=0.90)	Bootstrap: 0.3662 (p=0.94)
		Sensitivity: 0.0856 (0.0824, 0.0957)	Sensitivity: 0.3661 (0.3660, 0.3752)
Cheese intake	$E_{1}$: −0.0627	$E_{2}$: −0.0077	Direct: 0.3660
	(CI: −0.0762, −0.0493)	(CI: −0.0280, 0.0124)	(CI: 0.3527, 0.3793)
	Placebo: −0.0005 (p=0.94)	Indirect = 0.0005	Total = 0.3655
	Subset: −0.0628 (p=0.96)	Placebo: 0.0009 (p=0.88)	Placebo: −0.0004 (p=1.00)
	Bootstrap: −0.0612 (p=0.98)	Subset: −0.0083 (p=0.86)	Subset: 0.3658 (p=0.96)
	Sensitivity: −0.0627 (−0.0627, −0.0620)	Bootstrap: −0.0061 (p=0.94)	Bootstrap: 0.3672 (p=0.88)
		Sensitivity: −0.0077 (−0.0076, −0.0020)	Sensitivity: 0.3660 (0.3648, 0.3796)

Mediator	Loneliness → Mediator ($E_{1}$)	Mediator → T2DM ($E_{2}$)	Loneliness → T2DM (Direct/Total)
Processed meat	$E_{1}$: 0.0270	$E_{2}$: 0.0902	Direct: 0.3652
	(CI: 0.0060, 0.0480)	(CI: 0.0766, 0.1038)	(CI: 0.3488, 0.3817)
	Placebo: 0.0015 (p=0.90)	Indirect = 0.0024	Total = 0.3676
	Subset: 0.0273 (p=1.00)	Placebo: −0.0011 (p=0.92)	Placebo: −0.0001 (p=0.94)
	Bootstrap: 0.0275 (p=0.94)	Subset: 0.0903 (p=0.96)	Subset: 0.3652 (p=0.96)
	Sensitivity: 0.0270 (0.0271, 0.0280)	Bootstrap: 0.0904 (p=0.92)	Bootstrap: 0.3657 (p=0.98)
		Sensitivity: 0.0902 (0.0887, 0.0934)	Sensitivity: 0.3652 (0.3619, 0.3679)
Salt added	$E_{1}$: 0.0104	$E_{2}$: 0.1147	Direct: 0.3652
	(CI: −0.0053, 0.0260)	(CI: 0.0942, 0.1353)	(CI: 0.3488, 0.3817)
	Placebo: 0.0001 (p=0.88)	Indirect = 0.0001	Total = 0.3653
	Subset: 0.0102 (p=0.94)	Placebo: −0.0003 (p=0.96)	Placebo: −0.0016 (p=0.90)
	Bootstrap: 0.0118 (p=0.92)	Subset: 0.1153 (p=0.92)	Subset: 0.3656 (p=0.94)
	Sensitivity: 0.0104 (0.0094, 0.0104)	Bootstrap: 0.1136 (p=1.00)	Bootstrap: 0.3653 (p=1.00)
		Sensitivity: 0.1147 (0.1132, 0.1211)	Sensitivity: 0.3652 (0.3653, 0.3697)
Sugary cereals	$E_{1}$: 0.0300	$E_{2}$: 0.0755	Direct: 0.3629
	(CI: 0.0145, 0.0455)	(CI: 0.0584, 0.0960)	(CI: 0.3488, 0.3817)
	Placebo: 0.0006 (p=0.98)	Indirect = 0.0023	Total = 0.3652
	Subset: 0.0302 (p=0.98)	Placebo: −0.0007 (p=0.94)	Placebo: 0.0001 (p=0.96)
	Bootstrap: 0.0296 (p=0.98)	Subset: 0.0752 (p=0.88)	Subset: 0.3650 (p=0.90)
	Sensitivity: 0.0300 (0.0298, 0.0303)	Bootstrap: 0.0752 (p=0.96)	Bootstrap: 0.3642 (p=1.00)
		Sensitivity: 0.0755 (0.0744, 0.0790)	Sensitivity: 0.3652 (0.3633, 0.3734)
Cheese intake	$E_{1}$: −0.0258	$E_{2}$: −0.0233	Direct: 0.3642
	(CI: −0.0422, −0.0117)	(CI: −0.0428, −0.0049)	(CI: 0.3493, 0.3793)
	Placebo: 0.0004 (p=0.94)	Indirect = 0.0006	Total = 0.3648
	Subset: −0.0258 (p=0.94)	Placebo: −0.0022 (p=0.78)	Placebo: 0.0000 (p=0.94)
	Bootstrap: −0.0263 (p=0.96)	Subset: −0.0232 (p=0.98)	Subset: 0.3635 (p=0.78)
	Sensitivity: −0.0258 (−0.0281, −0.0261)	Bootstrap: −0.0242 (p=0.84)	Bootstrap: 0.3648 (p=0.94)
		Sensitivity: −0.0233 (−0.0302, −0.0170)	Sensitivity: 0.3642 (0.3618, 0.3664)

**Table 6:** Impact of mental health (anxiety/depression) on dietary behavior and T2DM risk: estimates and robustness checks. $E_{1}$ denotes the effect of the psychosocial exposure on the dietary mediator, $E_{2}$ denotes the effect of the mediator on T2DM risk, and indirect effects are computed as $E_{1}\times E_{2}$. Direct effects represent controlled direct effects of the exposure on T2DM, and total effects are the sum of direct and indirect components. All effects are reported on the probability scale as ATT. Confounders were selected from the DAG: age, long-term condition, and ethnicity. For psychosocial exposures, the other two stressors were included to block shared causes. In $E_{2}$ models, the exposure and non-focal diet indicators were added to reduce residual confounding. Direct-effect models conditioned on the mediator to estimate controlled direct effects. Refuters served as robustness checks rather than conventional confidence intervals. Sensitivity entries report refutation diagnostics rather than conventional confidence intervals.

Mediator	Mental health → Mediator ($E_{1}$)	Mediator → T2DM ($E_{2}$)	Mental health → T2DM (Direct/Total)
Processed meat	$E_{1}$: 0.0298	$E_{2}$: 0.0936	Direct: 0.3494
	(CI: 0.0016, 0.0579)	(CI: 0.0801, 0.1070)	(CI: 0.3275, 0.3714)
	Placebo: −0.0004 (p=0.96)	Indirect = 0.0028	Total = 0.3522
	Subset: 0.0304 (p=0.90)	Placebo: −0.0001 (p=1.00)	Placebo: 0.0006 (p=0.94)
	Bootstrap: 0.0290 (p=0.96)	Subset: 0.0930 (p=0.90)	Subset: 0.3490 (p=0.96)
	Sensitivity: 0.0298 (0.0270, 0.0289)	Bootstrap: 0.0936 (p=0.98)	Bootstrap: 0.3493 (p=0.92)
		Sensitivity: 0.0936 (0.0950, 0.0999)	Sensitivity: 0.3494 (0.3498, 0.3641)
Salt added	$E_{1}$: 0.0391	$E_{2}$: 0.1282	Direct: 0.3501
	(CI: 0.0183, 0.0599)	(CI: 0.1080, 0.1484)	(CI: 0.3281, 0.3720)
	Placebo: 0.0015 (p=0.86)	Indirect = 0.0050	Total = 0.3551
	Subset: 0.0391 (p=0.96)	Placebo: −0.0007 (p=0.92)	Placebo: 0.0016 (p=0.94)
	Bootstrap: 0.0392 (p=1.00)	Subset: 0.1287 (p=0.92)	Subset: 0.3500 (p=0.90)
	Sensitivity: 0.0391 (0.0389, 0.0397)	Bootstrap: 0.1291 (p=0.98)	Bootstrap: 0.3502 (p=0.98)
		Sensitivity: 0.1283 (0.1253, 0.1311)	Sensitivity: 0.3501 (0.3438, 0.3535)
Sugary cereals	$E_{1}$: 0.0198	$E_{2}$: 0.0999	Direct: 0.3501
	(CI: −0.0007, 0.0404)	(CI: 0.0804, 0.1196)	(CI: 0.3281, 0.3720)
	Placebo: 0.0009 (p=0.92)	Indirect = 0.0020	Total = 0.3521
	Subset: 0.0193 (p=0.94)	Placebo: 0.0005 (p=0.92)	Placebo: −0.0003 (p=0.96)
	Bootstrap: 0.0198 (p=0.90)	Subset: 0.0997 (p=0.92)	Subset: 0.3498 (p=0.96)
	Sensitivity: 0.0198 (0.0197, 0.0201)	Bootstrap: 0.0986 (p=0.80)	Bootstrap: 0.3494 (p=0.92)
		Sensitivity: 0.0999 (0.0958, 0.1022)	Sensitivity: 0.3501 (0.3487, 0.3521)
Cheese intake	$E_{1}$: −0.0102	$E_{2}$: −0.0427	Direct: 0.3501
	(CI: −0.0308, 0.0103)	(CI: −0.0636, −0.0219)	(CI: 0.3281, 0.3720)
	Placebo: 0.0007 (p=0.86)	Indirect = 0.0004	Total = 0.3505
	Subset: −0.0111 (p=0.86)	Placebo: −0.0003 (p=0.90)	Placebo: 0.0002 (p=0.94)
	Bootstrap: −0.0102 (p=0.92)	Subset: −0.0425 (p=0.94)	Subset: 0.3499 (p=0.98)
	Sensitivity: −0.0102 (−0.0118, −0.0102)	Bootstrap: −0.0436 (p=0.96)	Bootstrap: 0.3506 (p=0.98)
		Sensitivity: −0.0427 (−0.0431, −0.0375)	Sensitivity: 0.3501 (0.3420, 0.3547)

On the other hand, dietary mediation provided a smaller, reinforcing contribution within the estimated causal pathways. For instance, for *insomnia* the largest single mediated path was via salt (indirect ≈0.005), followed by processed meat (≈0.004) and sugary cereals (≈0.003); the cheese path was near zero and directionally harmful, reflecting reduced intake of a protective food under stress. For *loneliness*, processed meat and sugary cereals each mediated ≈0.002−0.0025, salt was negligible (≈0.0001), and cheese again showed a near-zero, directionally harmful path for the same reason. For *mental health*, salt was the main mediator (≈0.005), with smaller contributions from processed meat (≈0.003) and sugary cereals (≈0.002), and cheese followed the same minimal, harmful trend.

Summing across mediators, indirect effects through diet mediated about 0.5–1.3 percentage points of absolute risk for each stressor (roughly ∼1%–4% of the corresponding total effect), illustrating a consistent pattern within the observed data by which psychosocial stress is associated with unhealthier eating behaviors. Greater consumption of processed, salty, and sugary foods, together with lower intake of protective foods such as cheese, appears to further reinforce the estimated diabetes risk associated with psychosocial stressors within this cohort. The robustness of both direct and mediated pathways was confirmed through placebo, subset, bootstrap, and sensitivity refutation tests, with generally high p-values (mostly above 0.84, a few between 0.66 and 0.83), supporting the internal stability of these findings under the specified modeling assumptions.

### 10.3 Q3. Combined psychosocial stressors and T2DM risk

In real-world settings, psychosocial stressors rarely occur in isolation. Many individuals experience insomnia, loneliness, and poor mental health simultaneously, amplifying T2DM risk. To capture this clustering, a binary combined-stressor variable was defined, coded 1 when psychiatrist contact, loneliness, and insomnia co-occurred and 0 otherwise. The reported direct effect is the joint controlled direct effect (ATT) of this cluster, rather than the sum of individual stressor effects. As summarized in Table 7, the clustering of these stressors produced an ATT for the direct effect of 0.7796 (95% CI: 0.7457 to 0.8135), i.e., a 77.96 percentage-point increase in absolute risk; the corresponding total effect ATT was 0.8044. This direct effect is more than double the 0.35–0.37 risk differences (35–37 percentage points) linked to individual stressors. Balance and overlap were satisfactory: propensity score distributions showed good common support, all fitted risks lay within [0,1], and post-matching covariate balance met standard thresholds (all absolute standardized mean differences <0.1). These results indicate that, within this study population, co-occurring stressors are associated with substantially higher estimated diabetes risk than any single stressor alone.

**Table 7:** Impact of simultaneous psychosocial stressors on dietary behavior and T2DM risk: estimates and robustness checks. The combined stressor equals 1 when psychiatrist contact (consulted a doctor for anxiety or depression), loneliness, and insomnia co-occur. $E_{1}$ denotes the effect of the combined stressor on the dietary mediator, $E_{2}$ denotes the effect of the mediator on T2DM risk, and indirect effects are computed as $E_{1}\times E_{2}$. Direct effects represent controlled direct effects of the combined stressor on T2DM, and total effects are the sum of direct and indirect components. All effects are reported on the probability scale as ATT. Confounders were selected from the causal DAG and include age, ethnicity, and long-term condition; $E_{2}$ models additionally adjust for non-focal dietary indicators where applicable. Sensitivity entries report refutation diagnostics rather than conventional confidence intervals.

Mediator	Psych-stressors → Mediator ($E_{1}$)	Mediator → T2DM ($E_{2}$)	Psych-stressors → T2DM (Direct/Total)
Processed meat	$E_{1}$: 0.0704	$E_{2}$: 0.1530	Direct: 0.7796
	(CI: 0.0018, 0.1389)	(CI: 0.1395, 0.1666)	(CI: 0.7457, 0.8135)
	Placebo: 0.0048 (p=1.00)	Indirect = 0.0108	Total = 0.7904
	Subset: 0.0730 (p=0.94)	Placebo: 0.0008 (p=0.84)	Placebo: 0.0040 (p=0.88)
	Bootstrap: 0.0702 (p=0.92)	Subset: 0.1532 (p=0.98)	Subset: 0.7798 (p=1.00)
	Sensitivity: 0.0704 (0.0686, 0.0737)	Bootstrap: 0.1542 (p=0.94)	Bootstrap: 0.7814 (p=0.90)
		Sensitivity: 0.1530 (0.1484, 0.1539)	Sensitivity: 0.7796 (0.7605, 0.7726)
Salt added	$E_{1}$: 0.1352	$E_{2}$: 0.1831	Direct: 0.7796
	(CI: 0.0880, 0.1823)	(CI: 0.1617, 0.2044)	(CI: 0.7457, 0.8135)
	Placebo: −0.0005 (p=0.98)	Indirect = 0.0248	Total = 0.8044
	Subset: 0.1354 (p=0.94)	Placebo: −0.0009 (p=0.88)	Placebo: −0.0010 (p=1.00)
	Bootstrap: 0.1391 (p=0.98)	Subset: 0.1837 (p=1.00)	Subset: 0.7804 (p=0.94)
	Sensitivity: 0.1352 (0.1349, 0.1407)	Bootstrap: 0.1829 (p=0.98)	Bootstrap: 0.7825 (p=0.94)
		Sensitivity: 0.1831 (0.0807, 0.1865)	Sensitivity: 0.7796 (0.7698, 0.7853)
Sugary cereals	$E_{1}$: 0.1167	$E_{2}$: 0.1024	Direct: 0.7796
	(CI: 0.0680, 0.1654)	(CI: 0.0846, 0.1209)	(CI: 0.7457, 0.8135)
	Placebo: −0.0003 (p=0.96)	Indirect = 0.0120	Total = 0.7916
	Subset: 0.1149 (p=0.96)	Placebo: −0.0003 (p=0.98)	Placebo: 0.0014 (p=0.98)
	Bootstrap: 0.1167 (p=0.98)	Subset: 0.1018 (p=0.88)	Subset: 0.7801 (p=1.00)
	Sensitivity: 0.1167 (0.1075, 0.1158)	Bootstrap: 0.1034 (p=0.94)	Bootstrap: 0.7866 (p=0.66)
		Sensitivity: 0.1024 (0.0887, 0.1017)	Sensitivity: 0.7796 (0.7791, 0.7865)
Cheese intake	$E_{1}$: −0.0259	$E_{2}$: −0.0058	Direct: 0.7796
	(CI: −0.0801, 0.0283)	(CI: −0.0266, 0.0151)	(CI: 0.7457, 0.8135)
	Placebo: 0.0038 (p=0.88)	Indirect = 0.0002	Total = 0.7798
	Subset: −0.0242 (p=0.82)	Placebo: −0.0001 (p=0.94)	Placebo: 0.0028 (p=0.90)
	Bootstrap: −0.0274 (p=0.96)	Subset: −0.0053 (p=0.88)	Subset: 0.7808 (p=0.92)
	Sensitivity: −0.0259 (−0.0275, −0.0199)	Bootstrap: −0.0069 (p=0.88)	Bootstrap: 0.7843 (p=0.88)
		Sensitivity: −0.0058 (−0.0064, 0.0028)	Sensitivity: 0.7796 (0.7698, 0.7845)

Dietary mediation provided a smaller, reinforcing contribution. The largest single mediated path was via salt (indirect ≈0.0248, ∼3% of that model’s total effect, 0.8044). Sugary cereals and processed meat each added modest mediation (indirect ≈0.0120 and 0.0108, ∼1%–2% of their respective totals: 0.7916 and 0.7904). The cheese pathway was negligible and directionally harmful, reflecting reduced intake of a protective food (indirect ≈0.0002; total effect 0.7798). In short, within the modeled framework, clustered psychosocial stressors are associated with large direct increases in estimated T2DM risk, with diet contributing additional but comparatively small mediated increments.

Although indirect effects through diet explained only about 4.8 percentage points of the total risk (approximately 6%), their consistency underscores that dietary change is a meaningful pathway linking psychosocial stress with metabolic outcomes. Under the modeled counterfactual scenarios, this 2.5–4.8 percentage point diet-mediated component corresponds to an estimated reduction of 25,000–48,000 diabetes cases per million people, assuming similar risk structures, intervention uptake, and causal transportability. The robustness of both direct and mediated effects was confirmed through placebo, subset, bootstrap, and sensitivity refutation tests, where p-values were generally high, though a few fell below 0.88. Taken together, these findings emphasize that addressing stress, sleep disturbance, and poor mental health in tandem is critical, as combined stressors not only raise diabetes risk directly but also reinforce harmful dietary patterns that compound the metabolic burden.

#### 10.3.1 Discussion

This analysis reveals a consistent yet complex narrative: psychosocial stressors, including insomnia, loneliness, and mental health challenges, were associated with substantial increases in estimated T2DM risk, with direct physiological pathways accounting for the overwhelming majority of this effect (approximately 35 to 37 percentage points for single stressors, rising to nearly 78 percentage points for combined exposures). These direct pathways likely reflect well-established neuroendocrine and inflammatory responses to chronic stress, including heightened cortisol secretion, systemic inflammation, and dysregulated glucose metabolism [102–104]. Such biological effects underscore that psychosocial distress is not merely a mental or social challenge but a potent metabolic disruptor with real and measurable health consequences.

- **Dietary mediation: Subtle but consistent amplification:** Beyond these direct pathways, however, dietary mediation, though modest in absolute terms, emerges as a consistent, reproducible, and actionable contributor to T2DM risk. Across stressors, processed meat, salt, and sugary cereal intakes consistently appear as dietary amplifiers, suggesting a behavioral pivot toward energy-dense, ultra-processed foods in the face of stress [105]. Notably, insomnia-driven dietary shifts stand out as the largest indirect effect on dietary behaviors, particularly via salt intake, hinting at convenience eating driven by fatigue and disrupted circadian rhythms. Loneliness, in contrast, reveals a dietary profile characterized by reduced variety and lower intake of protective foods such as cheese and vegetables, patterns that may stem from disrupted social routines or diminished motivation to prepare balanced meals in isolation. Mental health challenges (e.g., depression, anxiety) also contribute to dietary disruption, though their effects on food intake appear smaller in magnitude compared to insomnia. This indicates that while mental health distress influences diet, sleep-related dietary shifts may be even more pronounced. It also suggests that dietary interventions which acknowledge emotional eating and low motivation, common in depression, are more likely to succeed than generic nutritional advice [106].
- **Mechanistic and structural explanations:** Beyond behavioral factors, psychosocial stress appears to accelerate diabetes risk through both neuroendocrine and inflammatory pathways. Persistent activation of the hypothalamic–pituitary–adrenal (HPA) axis increases cortisol levels, which impairs glucose regulation and promotes fat accumulation around the abdomen [107]. When combined with poor dietary habits, these physiological changes can further worsen metabolic health. Stress also alters levels of ghrelin and leptin, key hormones that regulate hunger and satiety, potentially encouraging overeating and unhealthy food choices [108]. Notably, exposure to such stressors is not evenly distributed. Individuals from marginalized groups, defined by race, socioeconomic status, or job type, face higher levels of chronic stress, including insomnia, social isolation, and psychological distress. These findings underscore the need to align diabetes prevention strategies with broader efforts to address social and health inequities.
- **Compounding effects of multiple stressors:** The interplay between psychosocial stressors becomes particularly striking when they co-occur. Combined stressor exposure not only amplifies direct diabetes risk well beyond the effect of any single stressor, a finding that suggests synergistic rather than additive physiological stress responses, but also intensifies dietary mediation. For example, under combined stressors, salt intake alone mediated over 3% of the total diabetes risk, doubling the mediation effect seen in single stressor scenarios. Sugary cereals and processed meat contributed additional increments of about 1%–2%, while reduced intake of protective foods such as cheese produced a small but directionally harmful contribution. In plain terms, when people face one kind of stress, eating less cheese makes a small difference to their diabetes risk. But when several stresses pile up together, that cheese effect is almost lost, and the bigger problem is a shift toward more salty, sugary, and processed foods. This compounding pattern underscores the importance of recognizing that psychosocial stressors often cluster together in real-world settings, driving both metabolic dysregulation and behavioral vulnerabilities in tandem.
- **Public health relevance and robustness:** Despite dietary pathways explaining only 0.1 to 3% of the total diabetes risk, their consistency and robustness, confirmed through rigorous placebo, subset, bootstrap, and sensitivity analyses, highlight them as genuine intervention points rather than statistical artifacts. Even small mediation effects can translate into substantial public health impact when scaled to population levels. For instance, a 3 percentage point dietary mediation effect could equate to approximately 30,000 potentially preventable diabetes cases per million individuals under comparable assumptions if effectively targeted. This underscores the real-world relevance of these pathways, particularly in high risk or marginalized populations already bearing a disproportionate burden of psychosocial stress.

#### 10.3.2 Implications for clinical and policy action

These findings have potential clinical and policy implications. First, they suggest the value of an expanded view of diabetes prevention that moves beyond simplistic dietary advice to consider upstream psychosocial drivers of unhealthy eating. Within the context of this study, poor food choices appear less as isolated personal decisions and more as responses shaped by chronic stress, fatigue, and emotional burdens. Interventions focused solely on one stressor (e.g., sleep loss) or one behavior (e.g., diet) may under-perform if they ignore the structural and psychological roots of these behaviors. A more comprehensive model that integrates psychological, social, and behavioral supports may be better suited for addressing multimorbidity and psychosocial clustering in vulnerable populations.

#### 10.3.3 Pathways to integrated, stress-informed interventions

Integrated care models that combine dietary guidance with stress management, sleep hygiene, and mental health support may offer a promising approach. For example, sleep interventions could address not only sleep duration and quality but also related dietary vulnerabilities, such as cravings for salty and processed foods. Loneliness interventions might incorporate structured meal planning and social meals to strengthen community support and foster healthier eating habits. Mental health services could consider targeting emotional eating and motivational barriers, recognizing that psychological distress often shapes food choices.

This interplay of biology and behavior highlights the need for holistic, stress informed interventions. Public health and clinical strategies may benefit from moving beyond isolated dietary counseling to address the psychological and social roots of poor eating habits. Integrated models that include sleep hygiene, mental health support, and nutrition counseling are particularly important for individuals facing multiple stressors. Additionally, addressing structural determinants, such as housing instability, job strain, and social isolation, will be essential for reducing these interconnected pathways to diabetes risk in marginalized populations.

1. **Recognizing structural and socioeconomic determinants:** Equally important is the recognition of structural and socioeconomic factors that intersect with these psychosocial stressors. Housing instability, occupational demands, and economic insecurity can magnify both direct stress effects and unhealthy dietary patterns, creating a feedback loop that entrenches metabolic risk. Future work should explore how these structural determinants modulate the observed pathways, informing precision prevention strategies tailored to the realities of diverse subgroups.
2. **Conclusion: Toward a more holistic framework:** The findings of this study support consideration of a shift from individual dietary counseling toward systems-level solutions. Interventions should not only promote healthy eating but also address upstream determinants such as sleep quality, access to mental health care, and community infrastructure for social connectedness. Policies that reduce work-related sleep deprivation, ensure mental health service coverage, and build neighborhood-based nutrition support could potentially mitigate psychosocial stress–driven metabolic risk.
  Future research should further disaggregate these pathways across demographic subgroups to better understand how intersecting social identities shape stress–diet–diabetes dynamics. Importantly, integrating mental and behavioral health services into chronic disease prevention may offer a high-leverage opportunity to improve outcomes. By identifying specific dietary habits that mediate psychosocial risk, this work clarifies where targeted, realistic interventions might succeed, especially in resource-constrained or high-burden settings.
  Within this study, the co-occurrence of insomnia, loneliness, social isolation, and poor mental health was associated with a strong and consistent increase in estimated T2DM risk. While much of the effect appears to be physiological or psychosocial in origin, poor diet acts as a reliable behavioral mediator, particularly through processed and salty foods. Public health strategies may need to account for the interconnected nature of stress, diet, and chronic disease, and respond with integrated, person-centered interventions that reflect this complexity.

### 10.4 Q4: Impact of simultaneously improving psychosocial factors on T2DM risk

This analysis examines the potential impact of a modeled holistic intervention targeting multiple psychosocial and behavioral risk factors simultaneously. Specifically, this study assessed the effect of improved mental health (no history of psychiatric consultation for anxiety or depression), reduced loneliness, and absence of insomnia on the overall risk of developing T2DM. These improvements were encoded as a single composite variable, *”Improved-Psychological-health”*, to reflect a real-world, integrated preventive strategy.

As shown in Table 8, improved psychosocial health was associated with a strong and statistically robust estimated protective effect on T2DM. The ATT was −0.1161 (95% CI: −0.1284 to −0.1039), corresponding to an 11.6 percentage-point lower absolute risk. This finding underscores the potential value of holistic interventions that address mental health, sleep, and social connectedness together. Robustness checks supported the stability of the estimate. Placebo effects were negligible (0, p=0.92), subset analyses closely matched the main result (−0.1162, p=0.98), and bootstrap replicates yielded nearly identical effects (−0.1159, p=0.96). However, sensitivity analysis indicated that the protective estimate (−0.1161) remained within a plausible range of −0.1239 to 0.0315. Because this range crosses zero, the protective effect could be attenuated to null or even reversed under certain levels of unmeasured confounding. Thus, while the consistency across robustness tests supports improved psychosocial health as a protective factor, caution is warranted in the causal interpretation.

**Table 8:** Estimated causal effect of improved psychosocial health on T2DM risk. Improved psychosocial health is defined as the absence of insomnia, loneliness, and psychiatric consultation for anxiety or depression. Effects are reported on the probability scale as the ATT. Confounders selected from the causal DAG include age, ethnicity, and long-term condition. Refutation tests (placebo, subset, bootstrap, and sensitivity analyses) assess robustness; sensitivity entries report refutation diagnostics rather than conventional confidence intervals.

Confounders	Improved psych-stressors → T2DM	Refutation tests
		Placebo: 0 (p=0.92),
Ethnicity, Age,	Estimate: −0.1161	Subset: −0.1162 (p=0.98),
Long term condition	(−0.1284, −0.1039)	Bootstrap: −0.1159 (p=0.96),
		Sensitivity: −0.1161 (−0.1239, 0.0315)

#### 10.4.1 Broader implications and mechanisms

These findings are consistent with the growing recognition that mental health, social relationships, and sleep are interconnected determinants of metabolic health. Depression and loneliness have been independently linked to systemic inflammation and hypothalamic–pituitary–adrenal (HPA) axis dysregulation [11, 107], while insomnia exacerbates sympathetic nervous system activity and impairs glucose metabolism [109, 110]. Addressing these domains together likely yields synergistic benefits that surpass the additive effects of targeting them in isolation [111].

This integrative perspective aligns with the “syndemic” framework in public health [111], which emphasizes how co-occurring psychosocial risks reinforce chronic disease pathways. For instance, improved sleep may buffer against mood disturbances and social withdrawal, while better mental health can support adherence to sleep hygiene and healthier routines. Such interdependence underscores the importance of moving beyond siloed strategies in prevention efforts.

#### 10.4.2 Clinical and public health relevance

From a clinical perspective, these results suggest the value of considering routine screening for psychosocial stressors such as depression, loneliness, and insomnia into T2DM prevention and management. Effective, multifaceted interventions could include:

- **Integrated behavioral counseling** that simultaneously addresses mood, social connectedness, and sleep hygiene.
- **Community-based programs** that reduce isolation and strengthen support networks, particularly for older adults and marginalized populations.
- **Collaborative care models** that unify primary care, mental health, and sleep medicine expertise into cohesive prevention strategies.

On a broader scale, these findings support upstream, population-level approaches. Policies that enhance social cohesion, expand access to mental health services, and improve sleep environments could collectively contribute to reducing the population burden of diabetes, pending validation in diverse real-world settings.

## 11 Limitations

Several limitations should be considered when interpreting the findings of this study. First, the analysis is based on a retrospective observational dataset, which limits causal identification to the assumptions underpinning the causal modeling framework. Although extensive covariate adjustment, expert-informed DAG construction, and multiple robustness checks were employed, the possibility of residual or unmeasured confounding cannot be fully excluded.

Second, several key exposures and mediators, including dietary behaviors, sleep disturbances, loneliness, and mental health indicators, were derived from self-reported data. Such measures may be subject to recall bias, reporting error, and misclassification, which could attenuate or inflate estimated effects. In addition, dietary variables were treated as baseline preferences rather than time-varying behaviors, limiting the ability to capture dynamic changes over the life course.

Third, modeling choices necessarily involved simplifications. Continuous variables such as BMI and dietary intake were dichotomized using clinically meaningful thresholds to facilitate causal interpretation and intervention simulation. While this improves interpretability, it may obscure nonlinear relationships or dose–response patterns. Similarly, psychosocial stressors were modeled as binary or composite exposures, which may not fully capture the severity or duration of these conditions.

Fourth, causal effect estimates rely on correct specification of the causal DAG and the assumption that all relevant confounders were observed and appropriately adjusted for. Although covariates were selected *a priori* based on domain knowledge, misspecification of causal relationships or omitted variables could bias estimates. The ATT estimates reported here reflect effects among the treated population and should not be interpreted as population-average effects or universal intervention impacts.

Finally, generalizability is limited. The findings reflect estimated counterfactual effects within the studied cohort and under the specified modeling assumptions. Population-level scaling and policy implications are illustrative and assume comparable risk structures, intervention uptake, and contextual factors. Prospective validation, incorporation of longitudinal and real-time data, and evaluation in diverse populations are needed before translating these findings into real-world intervention strategies.

## 12 Conclusion and future work

This study introduced a novel digital twin framework for predicting and simulating the onset of T2DM using retrospective behavioral, dietary, and psychosocial data. By moving beyond reliance on real-time monitoring and clinical biomarkers, the model demonstrates the potential for a low-burden, interpretable, and accessible approach to early disease prevention. Integrating psychosocial stressors such as insomnia, loneliness, and mental health history with dietary behaviors supported a more holistic characterization of estimated diabetes risk within this cohort. Key dietary factors, including processed meat, sugary cereals, salt intake, and cheese consumption, emerged as significant and modifiable predictors.

Causal inference analyses indicated that, under the modeled counterfactual assumptions, individual psychosocial stressors were associated with estimated increases in absolute T2DM risk of roughly 35 percentage points, while clustering of multiple stressors was associated with increases approaching 78 percentage points. Dietary mediation, though modest in absolute size, consistently reinforced these estimated effects within the modeled pathways, particularly through salt, processed foods, and reduced intake of protective foods. Importantly, the framework also revealed stark ethnic disparities, with individuals of Bangladeshi, Indian, Pakistani, African, and Caribbean descent showing substantially higher hazard ratios, highlighting the potential importance of culturally tailored prevention strategies. Validation through cross-validation (C-index = 0.90), placebo checks, bootstrap resampling, and sensitivity analyses supported the internal robustness of the modeling framework.

Building directly on the limitations identified in this study, future work will focus on extending the digital twin architecture beyond static, retrospective modeling. Incorporating longitudinal and real-time data streams, where available, will enable the representation of time-varying exposures, feedback mechanisms, and behavioral adaptation, addressing current constraints related to static counterfactual assumptions and self-reported measures. Such extensions would improve the realism of simulated interventions and allow the digital twin to evolve dynamically as individual risk profiles change over time.

Personalization of the framework could be further enhanced through the integration of genomic, geographic, and socioeconomic information, allowing more precise risk stratification and reducing residual confounding related to unobserved structural determinants of health. Expanding the framework to incorporate contextual factors such as healthcare access, neighborhood environments, and occupational stressors would further strengthen its relevance for diverse populations.

Ultimately, while the present findings are derived from modeled counterfactual scenarios within a specific cohort, this work lays the groundwork for future prospective validation and real-world evaluation. With further development and empirical testing, the digital twin framework has the potential to inform accessible, interpretable, and equitable prevention strategies that address both the biological and psychosocial dimensions of chronic disease risk.

## Data Availability

The data analyzed in this study is subject to the following licenses/restrictions: The UK Biobank dataset is not publicly available and can only be accessed by approved researchers through an application process. Requests to access these datasets should be directed to UkBiobank, https://www.ukbiobank.ac.uk/about-our-data/.
